# Identify and validate circadian regulators as potential prognostic markers and immune infiltrates in head and neck squamous cell carcinoma

**DOI:** 10.1038/s41598-023-46560-8

**Published:** 2023-11-15

**Authors:** Yi Jin, Zhanwang Wang, Siwei Huang, Chang Liu, Xiangwei Wu, Hui Wang

**Affiliations:** 1 Department of Radiation Oncology, Hunan Cancer Hospital, The Affiliated Cancer Hospital of Xiangya School of Medicine, Central South University, Changsha, 410013 Hunan China; 2 Key Laboratory of Translational Radiation Oncology, Department of Radiation Oncology, Hunan Cancer Hospital and The Affiliated Cancer Hospital of Xiangya School of Medicine, Central South University, Changsha, 410013 China; 3 Department of Oncology, Third Xiangya Hospital of Central South University, Changsha, 410013 China; 4 School of Humanities and Management, Hunan University of Chinese Medicine, Changsha, 410208 Hunan China; 5 Department of Nuclear Medicine, Hunan Cancer Hospital and The Affiliated Cancer Hospital of Xiangya School of Medicine, Central South University, Changsha, 410013 China

**Keywords:** Cancer, Biomarkers

## Abstract

Head and neck squamous cell carcinoma (HNSCC) is a heterogeneity pathological malignant cancer with leading causes of morbidity and mortality. EGFR inhibitors, immune checkpoint inhibitors have become novel treatments. However, the mechanism still remained uncertain. Several studies have confirmed that the circadian rhythms induce multiple malignancies developing. We utilized multi-omics analysis to demonstrate the crosstalk between circadian clock genes and tumor microenvironment in HNSCC. Firstly, we performed the LASSO Cox regression analysis based on the 16 important clock genes. A 7-gene risk model was successfully established in TCGA and validated in GEO datasets. Next, CIBERSORT and ESTIMATE methods were performed to display the immune landscape of high risk and low risk groups, and the results showed that high abundance of mast cells activated, dendritic cells activated and neutrophils were positively correlated with poor OS. To further identify hub genes, Kaplan Meier plot was applied in all TCGA and GEO datasets and two hub genes (PER2, and PER3) were identified, especially PER3, which was found strongly associated with immune score, PDCD1, CD4 + and CD8 + T cells in HNSCCC. Moreover, to explore the innate mechanism of circadian-induced pathway, we constructed a circadian-related ceRNA regulatory network containing 34 lncRNAs, 3 miRNAs and 4 core circadian genes. In-vitro experiments also verified that Per2 or Per3 could suppressed the proliferation, migration and invasion of HNSC. This study unraveled the association between PER3 and prognosis in patients with HNSC and the innate mechanism remains to be elucidated.

## Introduction

Head and neck squamous cell carcinoma (HNSCC) is a heterogeneity pathological malignant cancer, approximately 900,000 new cases in 2020^1^, which is the leading causes of morbidity and mortality worldwide^2^. The main treatment of HNSC is surgery or radiotherapy combined with chemotherapy and these therapies may reach a high expectation of cure while patients in early stages (I and II)^3^. Unfortunately, about 70% of patients were shown to have locally advanced cancer at the Initial diagnosis. The clinical outcome of multiple treatments was much less satisfactory with lower survival rate^4^. In recent years, EGFR inhibitors, immune checkpoint inhibitors have become novel anti-tumors treatments. However, this the therapeutic effect of cancer still remained uncertain while some patients not respond well^5^. Therefore, there is an urgent need to identify valuable clinical biomarkers to determine the biological behavior and prognosis of HNSCC, which will help improve therapeutic efficacy and prolong overall survival.

The endogenous 24-h oscillations—circadian rhythms, which could control daily behavior and biological processes. Sleep, blood pressure, body temperature, and blood hormone levels are depend on the circadian rhythm^6^. Circadian rhythms are generated from circadian genes that are regulated by biological clocks, which won the Nobel Prize in 2017^7^. In general, some biomarkers have been identified as core circadian clock genes^8,9^. It is well-known that the circadian regulatory feedback loops are comprised of CLOCK and ARNTL (BMAL1), and their downstream clock-controlled targets comprising Period genes (Per1, Per2, Per3) and Cryptochrome genes (Cry1 and Cry2). The CLOCK and ARNTL are activated in the morning, while the proteins of PER, CRY are accumulated and up-regulated to inhibit the CLOCK: ARNTL complex at night^10,11^.

Several researches in line with the epidemiological studies had demonstrated that circadian rhythms exhibit increased the susceptibility for developing multiple malignancies, including lymphoma, lung adenocarcinoma and breast cancer^12^. For example, BMAL2 was found to modulate intestinal stem cell pathways, such as Hippo signaling, and loss of circadian rhythms enhances tumor initiation^13,14^. The up-regulation of PER1 causes the reactivation of ATM-CHK2-P53/CDC25C signaling pathway and inhibits the growth of pancreatic cancer cells^15^. At present, the mechanism of circadian rhythm in HNSCC is not clear, and its significance in the treatment and prognosis of HNSCC needs to be further clarified.

Thus far, with the support of multi-omics analysis, we aim to answer the question of how the circadian clock shapes the tumor microenvironment of HNSCC. Through various bioinformatics tools and methods, the expression level and clinical significance of circadian clock genes were displayed in this study, moreover, we comprehensively demonstrated the crosstalk between biological clock and tumor microenvironment, providing new insights for individualized treatment of HNSCC.

## Materials and methods

### Data collection and processing

The RNA-sequencing of 16 clock genes (CRY1, CRY2, CLOCK, ARNTL, CSNK1D, CSNK1E, DBP, PER1, PER2, PER3, NR1D1, NR1D2, RORA, RORB, RSPO4 and SKP1) in HNSCC, including FPKM value of mRNAs, lncRNAs, miRNAs, as well as the corresponding clinical information were obtained from the The Cancer Genome Atlas (TCGA) website (https://cancergenome.nih.gov/)^16–20^. Total of 500 HNSCC samples were downloaded from TCGA database, and 10 patients lacking follow-up information or the survival was less than 30 days were excluded from survival analysis. In the validation set, the microarray list spectrum and clinical information are from the GEO database (ncbi. nlm.nih.gov/GEO/), and the accession numbers are GSE31056 and GSE65858. TCGA and GEO databases are common data repositories. This study follows the data access, publication policies and guidelines stipulated by TCGA and GEO databases.

### Prognosis model analysis and establishing

Combined with the gene expression and survival profiles of each sample, Least Absolute Shrinkage and Selection Operator (LASSO)-penalized Cox regression analysis was used to construct the risk model predicted by overall survival (OS). The risk score was calculated according to the circadian rhythm gene expression level, and the calculation formula was as follows: Risk score = expressed mRNA1 × coefficient mRNA1 + expressed mRNA2 × coefficient mRNA2 + expressed mRNAn × coefficient mRNAn. Patients were divided into high-risk and low-risk group with median score as the critical value. Survival analysis was applied to the “survminer” R package of training and validation sets showing differences in OS and progression-free survival (PFS) between different risk groups.

### HNSCC classification and enrichment analysis

Based on the risk groups, HNSC patients was classified as two clusters for further analysis. To verify the performance of the gene signature, we utilized the three ways to re-evaluate the classification, including NMDS, PCA and PLS_DA. Differentially expressed genes (DEGs) between high-risk and low-risk groups were calculated through ‘‘limma’’ R package. Annotation and visualization of GO terms was obtained from metascape (http://metascape.org/gp/index.html#/main/step1). Using GSEA software (v4.1.0) on GSEA web MSIGDB database (http://software.broadinstitute. org/gsea/msigdb) to analyze the gene functions.

### Immune infiltrate analysis

To evaluate immune environment of HNSCC, we uploaded data—gene expression matrix to CIBERSORT (https://cibersort.stanford.edu/) and visualized the correlation of 22 kinds of infiltrating immune cells. In addition, the presence of infiltrating immune cells and stromal cells was further assessed by an immune and stromal cell score calculated by an ESTIMATE algorithm using gene-level expression data (Estimate R package, v1.0.11).

### Cell culture and transfection

HNSCC cell lines (Cal-27) were purchased from American Type Culture Collection (ATCC), which cultured in DMEM (Invitrogen, New York, USA) containing 10% FBS (Procell, Wuhan, Hubei, China) and 1% penicillin–streptomycin (Procell, Wuhan, Hubei, China) at 37 °C and 5% CO_2_. SiRNAs targeting Per2 and Per3 and negative controls were synthesized by GenePharma Company (Suzhou, Jiangsu, China). The sequences of siRNAs were shown in Supplementary Table 2.

### Quantitative real time PCR (qRT-PCR)

Total RNA of Cal-27 cell was extracted through Trizol reagent (Invitrogen, USA) according to manufacturer's instructions. 1 µg of total RNA was reverse transcribed into cDNA through a PrimeScript RT Reagent Kit (Vazyme, Jiangsu, China), and a SYBR PCR Master Mix (Vazyme, Jiangsu, China) was used for qRT-PCR assay. Relative expression level was determined by utilizing the 2^−△△CT^ method with GAPDH as the internal control, and the sequences of primers used were exhibited in Supplementary Table 2.

### Cell counting Kit-8 assay

Cal-27 cell was plated into the 96-well plate at a density of 5000 cells/well, which were transfected with different siRNAs after seeding 16 h, and cells were cultured for the indicated time. 10 μl of Cell Counting Kit-8 (CCK-8) reagent (Biosharp, Anhui, China) were increased to a well, then the cells were incubated at 37 °C for 1.5 h. The optical density (OD) value was detected through a microplate reader (450 nm).

### EdU assay

In brief, Cal-27 cells (2.0 × 10^5^ cells/well) were seed into a 6-well plate, and the cells were transfected with NC or different siRNAs, then cells were cultured in an incubator for about 96 h. Cal-27 cells were incubated with EDU (Beyotime, Shanghai, China) for 3 h, and fixed with 4% of paraformaldehyde (Beyotime, Shanghai, China) for 30 min, then permeabilized with 0.3% Triton X-100 (Beyotime, Shanghai, China) for another 30 min. Next, 500-µL of the click reaction mixture was used to incubated the Cal-27 cells for 30 min, and cells were washed with 0.3% Triton X-100 three times and incubated with Hoechest buffer for 10 min.

### Colony formation assay

After transfection with different siRNAs for 24 h, the Cal-27 cells were counted and adjusted cells concentration to 1000 cells/well in 6-well plates. After incubation for 14 days, cell colony formation was stained and detected.

### Transwell assay

After transfected with different siRNAs for 24 h, Cal-27 cells were collected and washed with PBS for twice. 200 µL (1.0 × 10^5^ cells) of cell suspension diluted with serum-free DMEM was added into the upper chamber (Corning, USA), and 600 µL DMEM containing 20% FBS was added to the lower chamber. The cells were incubated at 37 °C for about 24 h. Finally, the infiltrated cells were stained with crystal violet buffer (Solarbio, Beijing, China) for 20 min, and the infiltrated cells were counted and photographed.

### Statistical analyses

All statistical analyses in this study were performed by using R software (v 3.6.1) and GraphPad Prism 9, and a *p* value < 0.05 was considered statistically significant for all analyses.

### Ethics approval and consent to participate

This study was approved by the Ethics Committee of Hunan Cancer Hospital.

## Results

### Construction of a prognostic risk model based on the circadian genes

**Figure 1:**
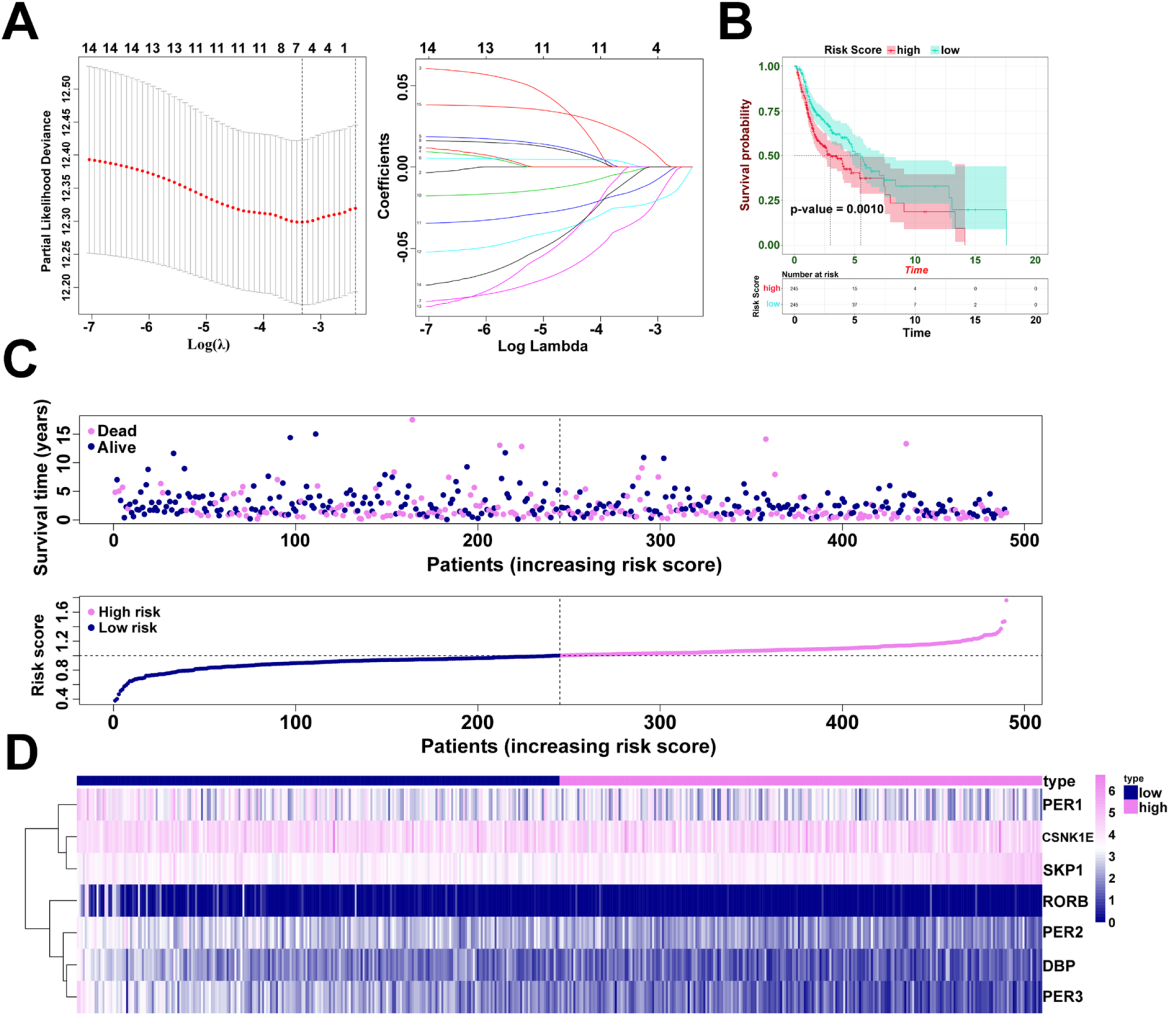
Circadian prognostic risk model construction. (**A**) Identification of clock genes from the LASSO Cox regression. (**B**) The Kaplan–Meier plot of OS between high and low risk groups. (**C**) Distributions of risk scores, survival status, and expression of high or low risk groups. (**D**) The heatmap of 7 circadian genes expression.

**Figure 2:**
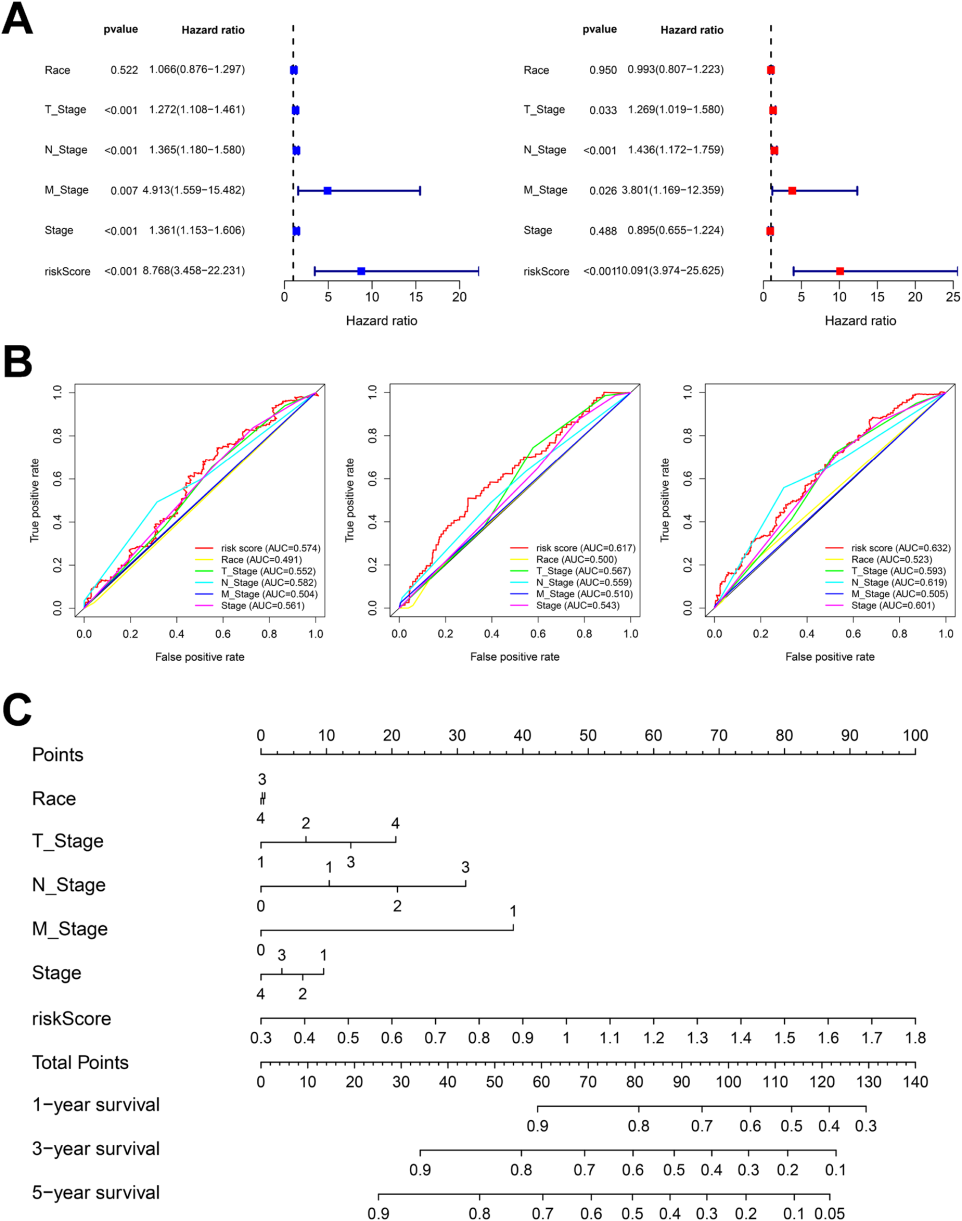
7-gene signature model evaluation. (**A**) Univariate (left) and multivariate (right) Cox regression analyses of clinical characteristics and risk score. (**B**) ROC curve of risk score and clinical characteristics in 1, 3 and 5 years. (**C**) A nomogram construction combined with risk score and clinical characteristics.

A total of 500 HNSC patients with OS were analyzed in TCGA dataset and 10 patients were excluded owing to the low OS (< 30 days). To establish a risk score system for predicting the survival time of patients with HNSCC, LASSO Cox regression analysis was performed to identify clock genes with strongest prognostic power (Fig. 1A). Consequently, 7 optimal genes (CSNK1E, DBP, PER1, PER2, PER3, RORB, and SKP1) were identified for establishing the risk model (Supplementary Table 1). Then, the patients with HNSCC were stratified into low-risk (245 patients) and high-risk groups (245 patients) based on the median risk score. To explore the prognostic role of the 7-gene signature model, we then conducted KM plot analysis for these two risk groups and found that the OS was significantly poor in patients from high-risk group compared to low-risk group (*p* = 0.001) (Fig. 1B). The distribution of risk scores and survival status of every patient is exhibited in Fig. 1C,D. We found specific genes were down-regulated in high risk group, especially the PER2 and PER3. In order to assess whether the risk score is independent of other clinical features as a prognostic factor of HNSCC, we conducted univariate and multivariate Cox regression analysis on TCGA cohort, and the results showed that the risk score was significantly correlated with patients’ OS (Fig. 2A and Supplementary Table 7) and have superior predictive value than pathological tumor, pathological nodes, and so on (Fig. 2B). Furthermore, we generated a nomogram for these independent risk factors (Fig. 2C). Each independent risk factor is assigned an associated score, resulting in an overall score. The total score helps predict one-, three- or five-year survival rate of patients with HNSCC, allowing us to estimate a patient's probability of survival more accurately and easily. Finally, the concordance of C-index was 0.649.

### Validation of the prognostic risk model and circadian biomarkers

**Figure 3:**
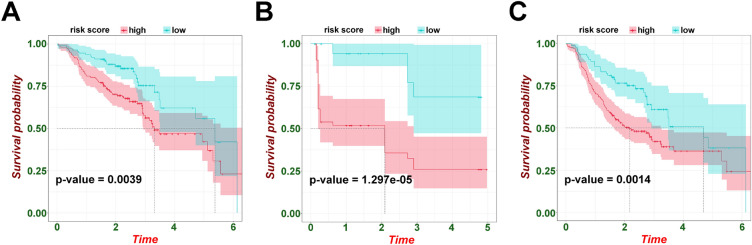
Prognostic signature validation. (**A**) The Kaplan–Meier plot of OS in GSE65858 based on this risk model. (**B**) The Kaplan–Meier plot of PFS in GSE65858 based on this risk model. (**C**) The Kaplan–Meier plot of PFS in GSE31056 based on this risk model.

The rigor of the model developed based on TGCA HNSCC cohort is verified in GEO datasets. In GSE65858, results showed a significantly higher chance of death (*p* = 0.0039) and a lower PFS time (*p* = 0.0014) in the high-risk group (Fig. 3A,B). In GSE31056, PFS deteriorated significantly in high-risk patients (Fig. 3C, p < 0.001). The prognostic analysis data of HNSCC core circadian rhythm genes were shown in Supplementary Fig. 1. In TCGA HNSC dataset, the higher expression of DBP, PER1, PER2, PER3 and RORB were linked to better OS, yet high expression of SKP1 was associated with poor OS (Supplementary Fig. 1A). Similarly, some crucial biomarkers were successfully re-verified in other GEO datasets (Fig. 1B–D).

### Tumor environment evaluation based on the HNSCC classification

**Figure 4:**
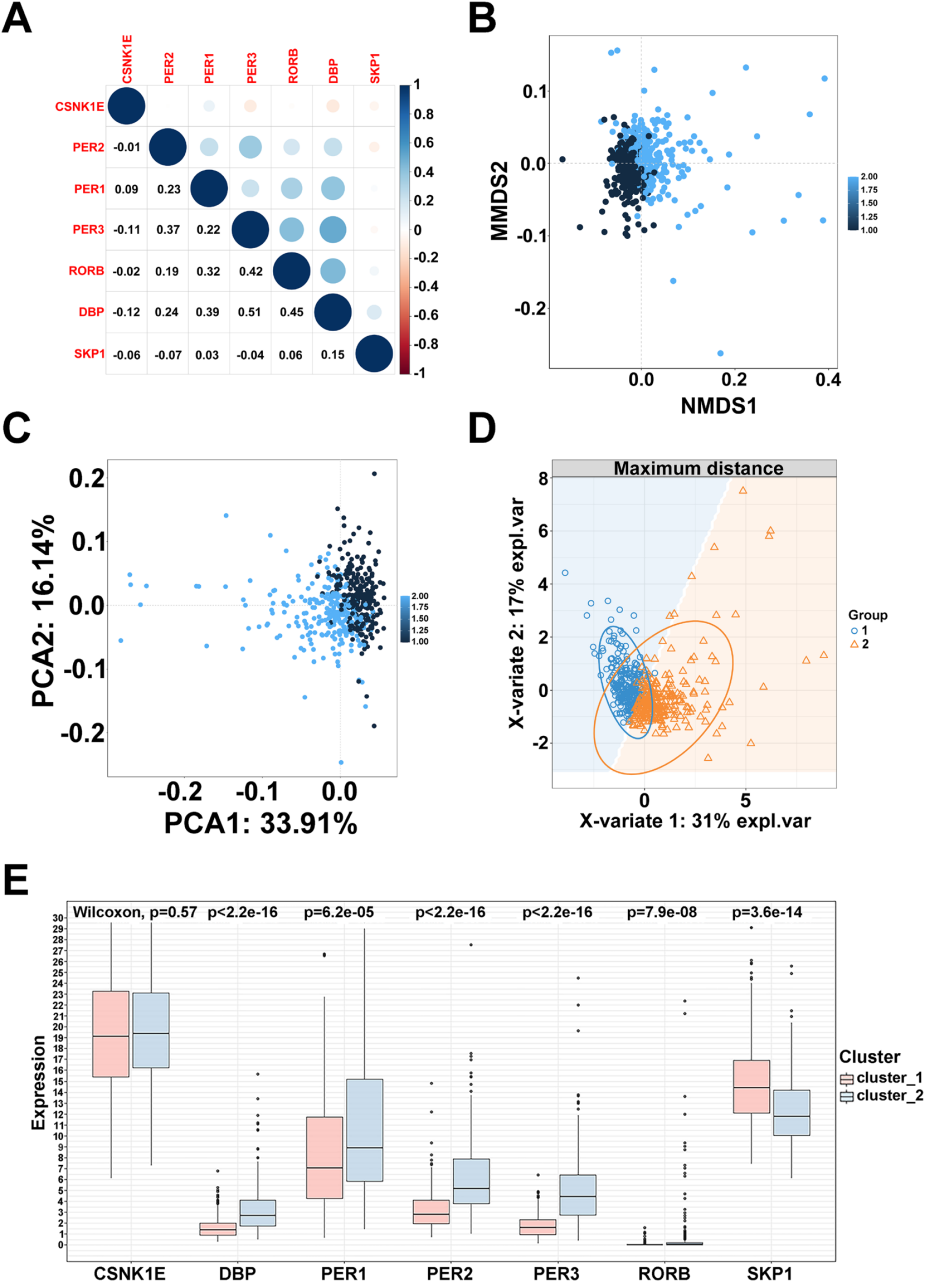
HNSC classification evaluation. (**A**) The good correlation among core circadian genes. (**B**) The methods of NMDS to examine the novel HNSC classification. (**C**) The methods of PCA to examine the novel HNSC classification. (**D**) The methods of PLS_DA to examine the novel HNSC classification. (**E**) The expression of 7 crucial clock biomarkers between high and low risk groups (***p < 0.001; **p < 0.01; *p < 0.05).

**Figure 5:**
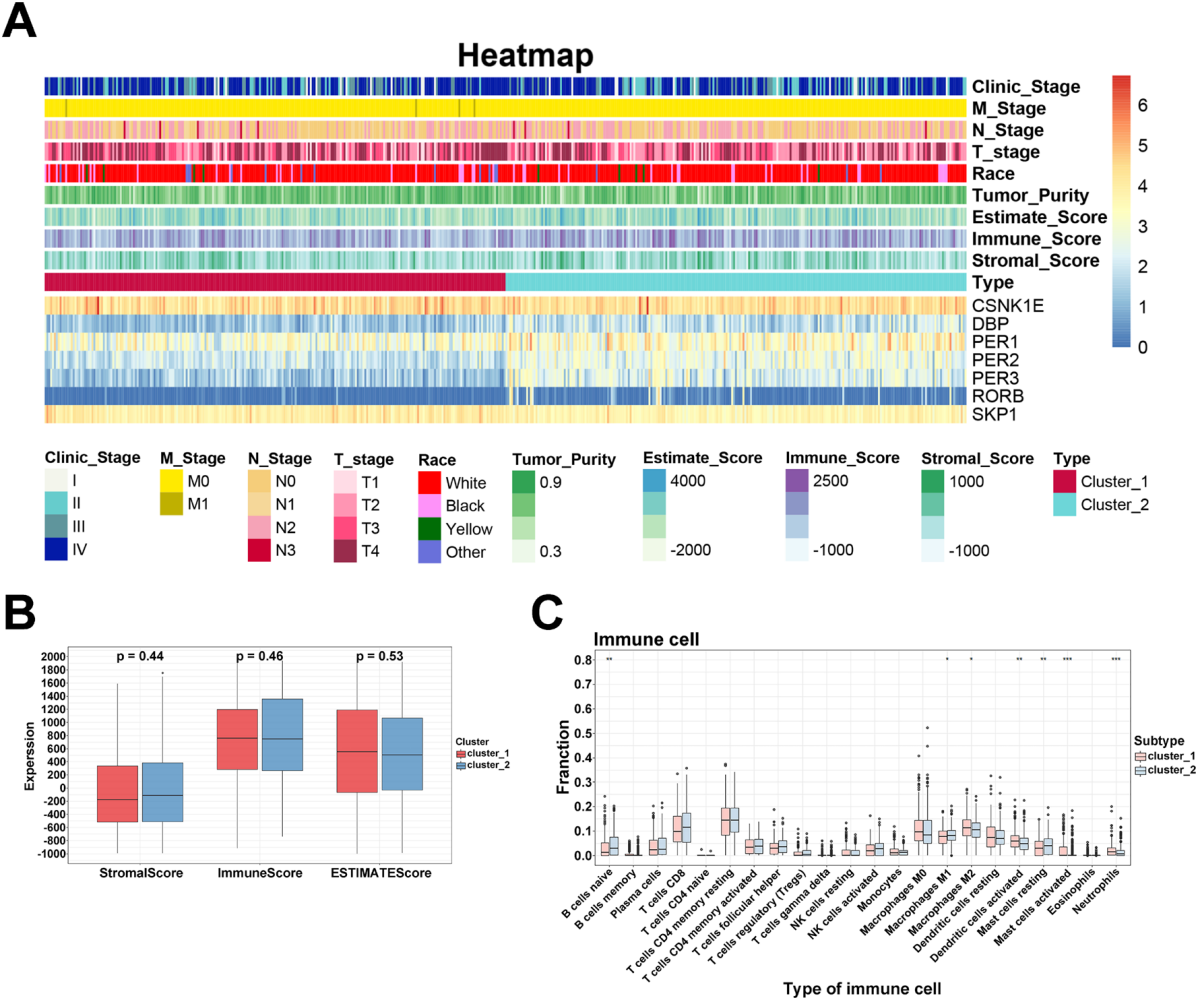
The correlation between the circadian and immune infiltration. (**A**) Heatmap of the expression of crucial genes, clinical characteristics and ESTIMATE scores. (**B**) Differential expression of stromal, immune, and ESTIMATE scores. (**C**) Differential distribution of 22 immune infiltrating cells in two clusters (***p < 0.001; **p < 0.01; *p < 0.05).

In TCGA HNSCC cohort, the plot showed the diverse correlations between core circadian genes (Fig. 4A). In current study, three methods of classification, NMDS, PCA and PLS_DA, calculated by the R packages ‘vegan’, ‘prcomp’,‘mixOmics’ respectively, were used to examine the novel HNSCC clusters (high risk/cluster 1 and low risk/cluster 2) on the basis of risk score. Apparently, all examination of three methods confirmed the high discrimination of HNSCC classification (Fig. 4B–D). Next, the crucial clock biomarkers at the mRNA level were shown in high and low risk groups (Fig. 4E). Immune infiltration is a vital factor affecting prognosis in HNSCC, to determine whether core circadian genes could be used as immune-therapeutic targets of HNSCC, we clarified the correlation between mRNA levels of these circadian genes and immune infiltration. Firstly, we visualized the distribution of core circadian genes, immune-related scores (ESTIMATE, immune, stromal score) and clinical factors in heatmap (Fig. 5A). Unfortunately, we failed to disclose the relationship between core circadian genes and immune-related scores (Fig. 5B). In addition, through the method of CIBERSORT, the high abundance of mast cells activated, dendritic cells activated and neutrophils were positively correlated with high-risk group with poor OS (*p* < 0.01), however, mast cells resting and B cells naïve were related to better OS (*p* < 0.01) (Fig. 5C).

### Functional enrichment in HNSCC classification

**Figure 6:**
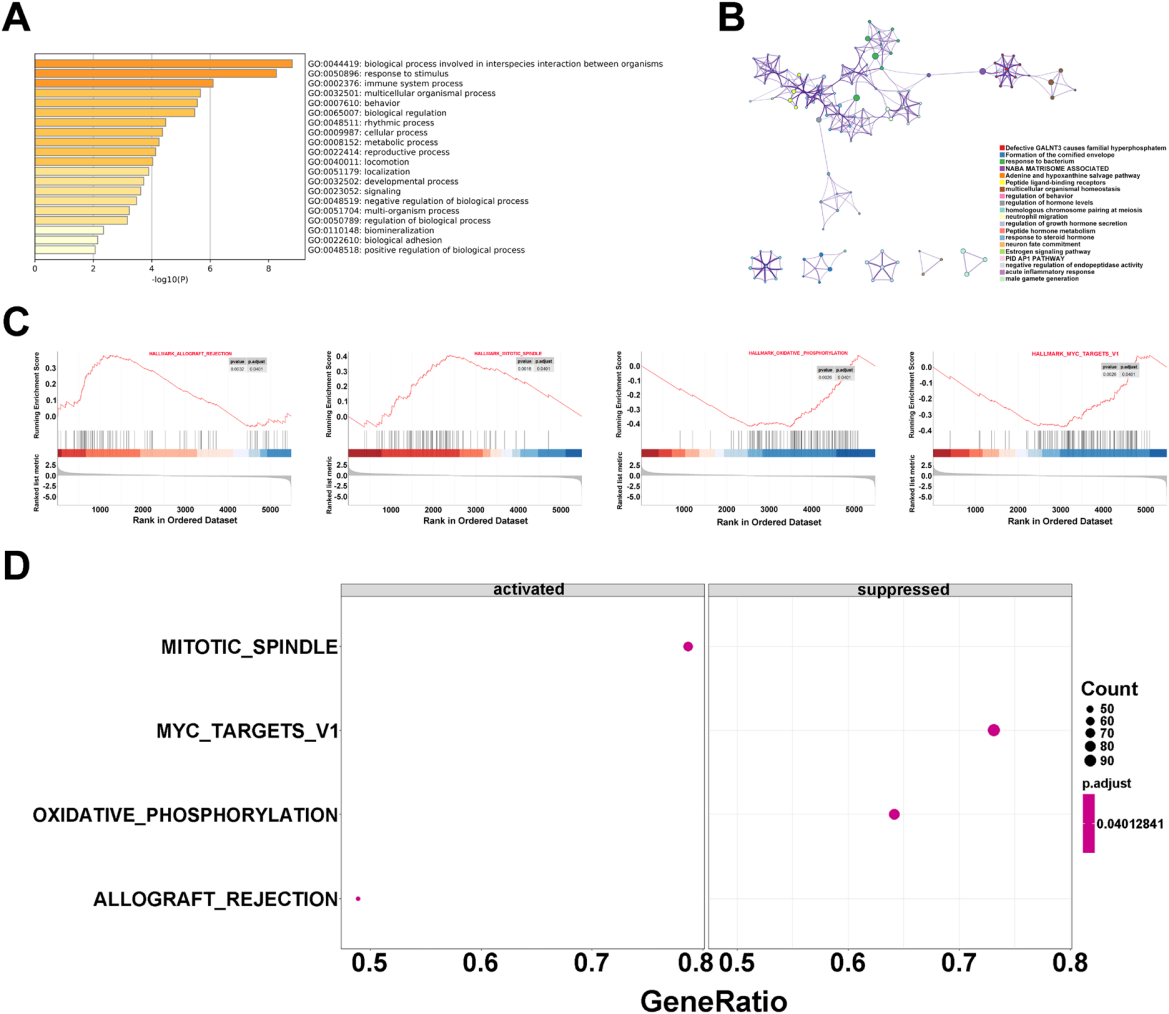
The innate mechanism in HNSC. (**A**,**B**) The visualization of classic pathways which DEGs enriched in. (**C**,**D**) Four functional pathways in development of HNSC through GSEA.

In this study, we calculated 5714 DEGs with* p* < 0.05 in two clusters (Cluster_1 and 2) and performed these DEGs on Metascape online tools. As shown in Fig. 6A, we found that the DEGs were mostly enriched in some classic pathways, like the formation of the cornified envelope, NABA matrisome associated, response to stimulus, immune system process, and rhythmic process of Gene Ontology (GO). In part of gene set enrichment analysis (GSEA), four functional pathways were stuck out to play a great role in development of HNSCC. Among those pathways, mitotic spindle and allograft rejection may activate the HNSCC tumorigenesis, however, MYC target v1 and oxidative phospho- rylation may suppress the trends of progression (Fig. 6B–D).

### Overview the hub circadian biomarkers in HNSCC

Considering the significance of the hub circadian gene in determining the prognosis value of patients with HNSCC, we combined the results of TCGA and GEO datasets estimated by the KM (Kaplan Meier) analysis. PER2, and PER3 were identified to be responsible for HNSCC malignancy and prognosis (Supplementary Fig. 1). Based on these above results, we studied the relationship between PER2, PER3 and immune infiltrating cells, which showed that the high expression of PER3 mRNA was positively correlated with the immune score (r = 0.16, *p* < 0.001), T cells regulatory (Tregs) levels (r = 0.10, *p* = 0.028), PDCD1 (r = 0.22, *p* < 0.001), T cells CD4 + memory activated (r = 0.10, *p* = 0.023), T cells CD8 + (r = 0.09, *p* = 0.036), T cells follicular helper (r = 0.12, *p* = 0.009) in HNSCC (Supplementary Fig. 2A). Thus, the relationship between PER3 mRNA expression and immune environment is worthy of attention. Unfortunately, there was no correlation between PER2 and immune characteristics (Supplementary Fig. 2B). The role of PER family in the malignancy of HNSCC was assessed. The MEXPRESS group was classified according to different clinical factors, including clinical T stage, pathologic T and N stage, HPV status, overall survival and copy number, PER3 displayed a differential expression pattern (Supplementary Fig. 3 and Table 3). Likewise, PER2 also presented diverse expression pattern of clinical M stage, histological type, HPV status, overall survival and copy number (Supplementary Fig. 4 and Table 4). The methylation level of PER3 and PER2 were shown in Supplementary Table 3 and S4. PER2 and PER3 were also founded to be significantly up-regulated in the normal tissue compared to the tumor tissue (Supplementary Fig. 5A,B). HPA database (https://www.proteinatlas.org/) was used to validate the protein level of candidate genes (Supplementary Fig. 5C,D).

### Exploration of mechanism based on the core circadian genes

**Figure 7:**
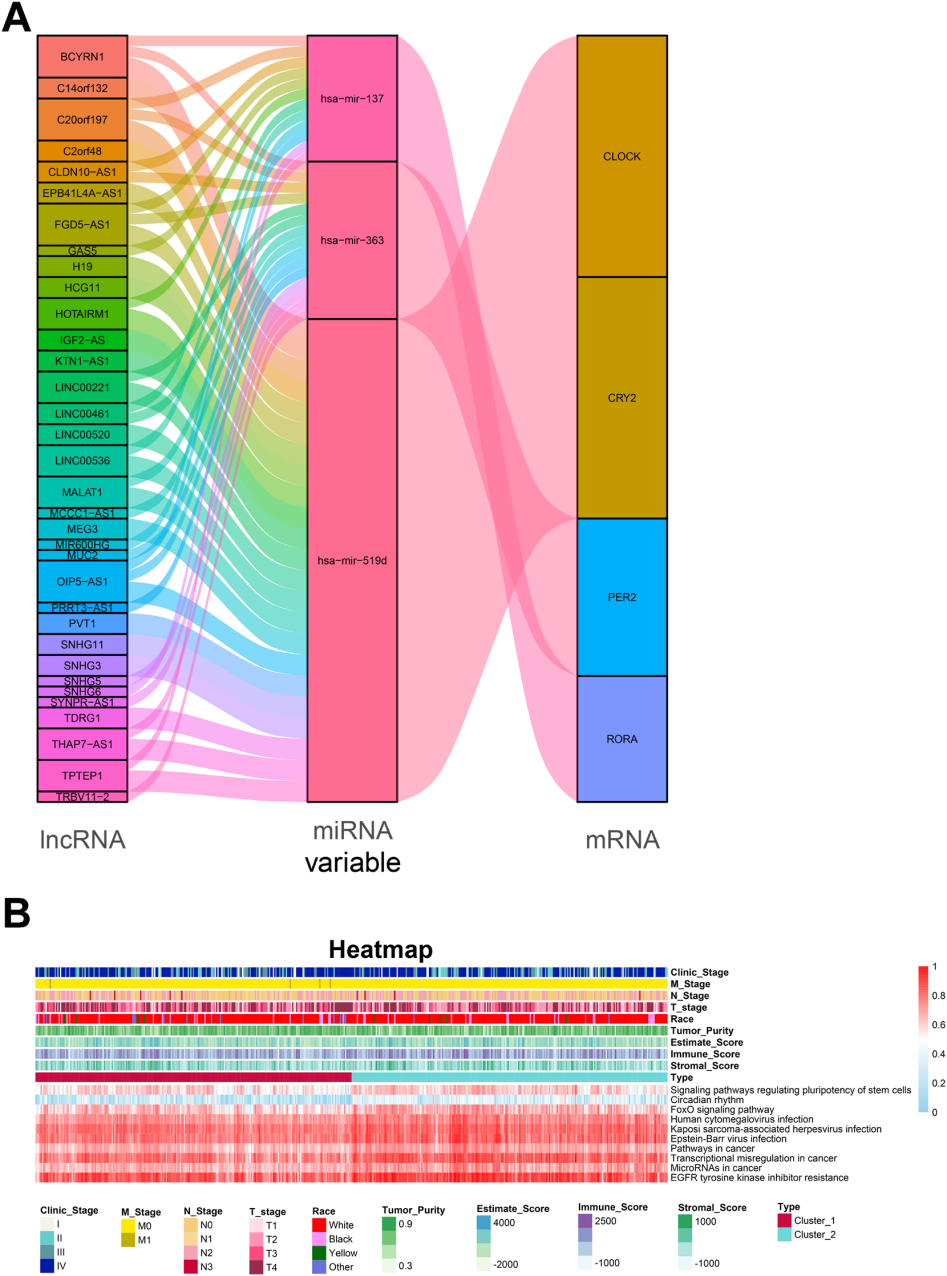
The construction of ceRNA based on the core circadian genes. (**A**) The circadian-related ceRNA regulatory network visualization. (**B**) Heatmap of the expression of signaling pathways, clinical characteristics and ESTIMATE scores.

To explore the innate mechanism of circadian-induced pathway combined with lncRNAs and miRNAs, we obtained 929 differentially expressed lncRNAs and 140 miRNAs by using TCGA database with *p* < 0.05 when in comparison with cluster 1 and 2. Based on four databases (miRcode, miRTarBase, miRDB, and TargetScan), we enrolled 59 lncRNAs and 9 miRNAs which targeted 503 mRNAs (Supplementary Table 5 and S6) and visualized a circadian-related ceRNA regulatory network containing 34 lncRNAs, 3 miRNAs and 4 core circadian genes (Fig. 7A). For further exploring the innate relationship among those circadian genes, we applied the KEGG analysis of 503 mRNAs and unravel 10 important functional signaling, such as circadian rhythm, signaling pathways regulating pluripotency of stem cells and FoxO signaling pathway. Furthermore, we employed the ssGSEA via R packages (GSVA, GSEABase, and limma) to comprehensively assess the signaling characteristics among each sample and drew a complex heatmap to depict the distribution of functional signaling, HNSC classification, immune score and clinical factors (Fig. 7B).

### Effect of Per2 and Per3 silencing on proliferation, migration and invasion of HNSCC

**Figure 8:**
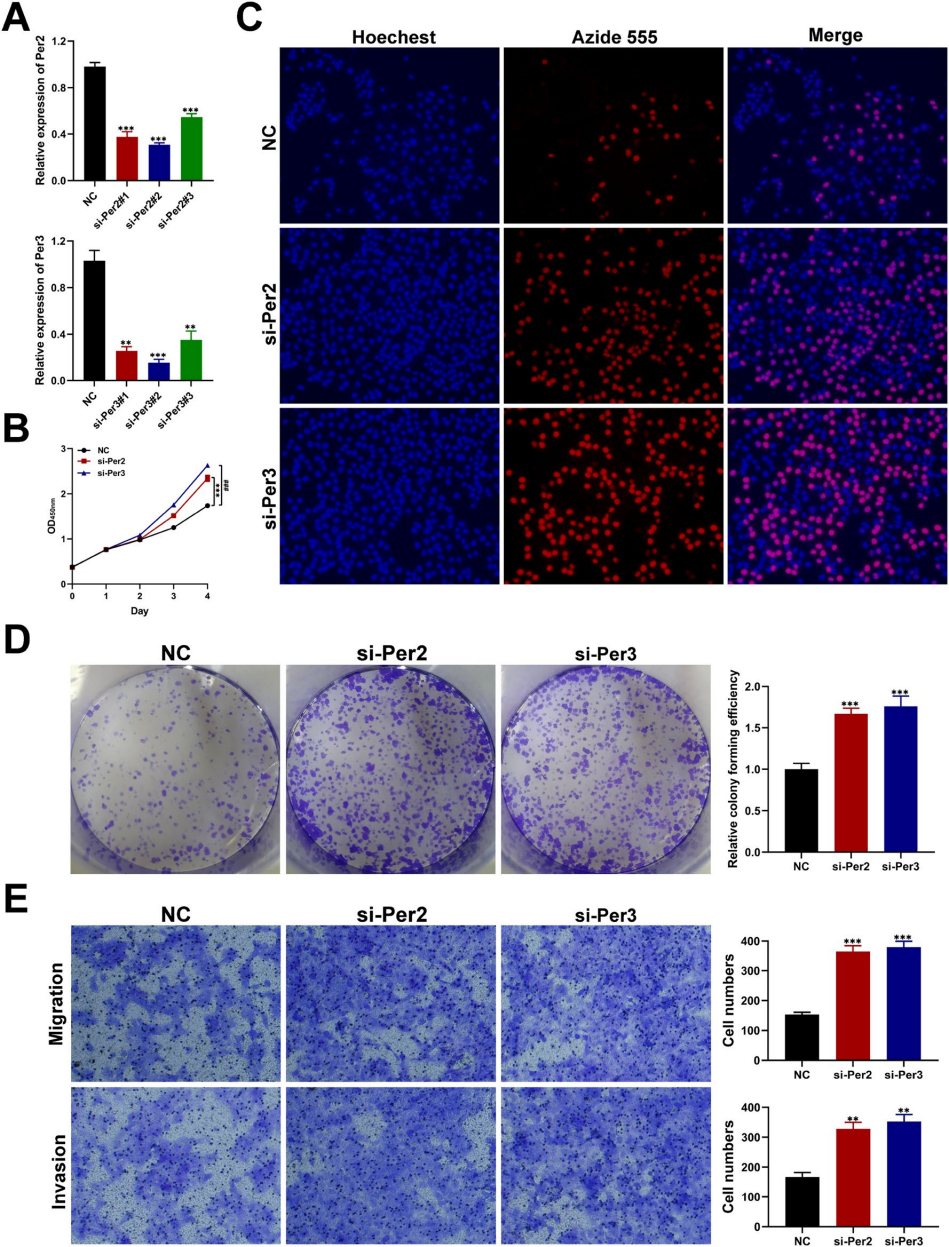
Biological functional validation of Per2 and Per3 in HNSC cells. (**A**) Silencing efficiency of Per2 and Per3. CCK8 (**B**), EDU (**C**) and colony formation assay (**D)** revealing the effect of Per2 or Per3 depletion on proliferation of HNSCC cells. (**E**) Transwell assay exhibiting that silencing of Per2 or Per3 significantly boosts the migration and invasion of HNSC cells (*p < 0.05, **p < 0.01, and ***p < 0.001, n = 3).

To verify the reliability of the diagnostic model we established, we selected Per2 and Per3 to further verified the vital role of the hub genes in HNSCC progression and development. We designed and synthesized siRNAs for Per2 and Per3 (named si-Per2#1, si-Per2#2, si-Per2#3 and si-Per3#1, si-Per3#2, si-Per3#3). qRT-PCR assay confirmed that the expression level of Per2 and Per3 was lower than the cells transfected with NC (Fig. 8A), and the depletion efficiency of si-Per2#2 (termed si-Per2) and si-Per3#2 (termed si-Per3) was highest so we chose them for further investigation. CCK8 and EDU assay verified that Per2 and Per3 depletion significantly boosted the growth rate of HNSCC cells (Fig. 8B,C). Furthermore, the proliferative rate of HNSCC cells was obviously promoted after silencing Per2 or Per3 by performing clone formation assay (Fig. 8D), which indicated that Per2 and Per3 play a vital role in the proliferation of HNSC. Transwell assay also revealed that Per2 or Per3 depletion strikingly enhanced the migration and invasion of HNSCC cells (Fig. 8E).

## Discussion

HNSCC are malignant tumors of the mouth, larynx and throat, which develop from squamous cells in the epithelial cells of the anatomical surface of the head and neck. The major risk factors of HNSCC included alcohol consumption, tobacco use, especially the human papilloma virus 16 (HPV16) infections^21^. Currently, lack of rapid improvement of survival rate and personalized treatment have propelled the study of HNSCC molecular map. However, the uncontrolled cell growth of HNSCC, is a complicated process coupled with multiple genetic mutation and tumorigenesis pathways^22,23^. Using expression arrays and, RNA sequencing, some molecular events in HNSCC are identified like epidermal growth factor receptor (EGFR), PD-1 (programmed cell death-1) or vascular endothelial growth factor (VEGF)^24–26^. Nevertheless, owing to the high intra-tumor heterogeneity of HNSCC, targeted therapy will yield limited solution. Most efficient treatments or molecular events observed are manifested to induce the DNA damage through oxidative reaction^27,28^. Interestingly, a more novel mechanism, circadian clock, a timing system within an organism, evolved through millions of years of evolutionary process, enables organisms to cope with and adapt to the daily light and dark cycle^29^, and may directly control some DNA repair mechanisms, including double strand or single strand break repair^30^. Therefore, we speculate that circadian rhythm may play an important role in the tumorigenesis of HNSCC.

Results from the enrichment analysis revealed that mitotic spindle, allograft rejection, MYC target and oxidative phosphorylation may take part in the tumorigenesis in HNSC coupled with circadian genes. Mitotic spindle is the essential process of cell cycle, and the failure of this spindle assembly checkpoint can result in aneuploidy and may be involved in the formation of cancer^31^. In HNSC, Dawei H observed that Aurora kinase A (AURKA) is a serine-threonine kinase that functions in mitotic spindle formation may up-regulate in HNSC cell lines and suppress the apoptosis rate and ROS generation in HNSCC possibly through the production of ROS^32^. Yet there was no literature to explore the relationship between mitotic spindle and circadian rhythm. In fact, patients with allograft rejection usually faced the high risk of cancer and the etiology of posttransplant malignant tumors is considered to be multifactorial, including impaired immune monitoring of tumor cells and decreased antiviral immune activity^33^. The MYC family are the most frequently deregulated driver genes in human cancer. Nowadays, theoretically, targeting MYC is an attractive strategy for cancer treatment. APTO-253 reduces the expression of MYC to a certain extent, and is currently conducting a phase I clinical trial in patients with relapsed/refractory acute myeloid leukemia or myelodysplastic syndrome^34^. Furthermore, MYC-associated factor X (MAX) plays a repressive role in binding to the core clock gene BMAL1 and maintaining a proper circadian rhythm^35^. In addition, some circadian rhythm genes are playing an essential role in reducing the development of solid tumors which switch cancer cells from aerobic glycolysis to mitochondrial oxidative phosphorylation^36^. Combining the literatures above, the great role of circadian rhythm in HNSCC may be verified in our study and multiple researches.

At present study, we observed multiple circadian genes, particularly PER2 and PER3, were downregulated in cancer tissue, likewise, Xiong also found that loss of PER2 expression was closely associated with the genesis and development of HNSCC^37^. Furthermore, Li validated the similar results by using real-time quantitative PCR (RT-PCR)^38^. Moreover, in our study, we found these two genes were protective biomarkers linked with better OS in HNSCC. However, there was rare literatures to explore the mechanism of PER family in HNSCC development. In cervical and esophageal cancer, overexpression of PER2 suppressed cell proliferation and activation of REV-ERBα and RORα resulted in cancer cell death^39^. In this study, we constructed a ceRNA network to depict the relationship among lncRNAs, miRNAs, and PER2. However, most relationship were not validated in vivo or vitro experiments. PER3 were identified as a most important protective indicator which superior to PER2. In a large GWAS meta-analyses, genetic variation of PER3 found to be significantly related with the risk of prostate cancer and lung cancer^18^. Mechanistically, PER3 negatively regulates stemness of prostate cancer stem cells via WNT/β-catenin signaling in the tumor microenvironment^40^. The results in our study displayed that high expression of PER3 were associated with essential immune cells or biomarkers like T cells CD4 + , T cells CD8 + and PD-L1, which providing a novel strategy to treat HNSCC patients. Likewise, in pan-renal cell carcinoma, PER3 was found to be positively correlated with the infiltration levels of CD4 + and CD8 + T cells^41^. In pan-cancer analysis, abnormal circadian clock contributes to T cell exhaustion and immune evasion^42^. Moreover, to further verify the accuracy and reliability of our diagnostic model, we performed in-vitro experiments and demonstrated that depletion of Per2 or Per3 obviously boosted proliferation, migration and invasion ability of HNSC cells, revealing that Per2 and Per3 are prognostic markers for HNSC. There are limitations to this study. For example, the present study is a retrospective study on the basis of publicly available databases which may make more selection bias.

## Conclusions

In conclusion, this study unraveled the association between PER2, PER3 and prognosis in patients with HNSC and the innate mechanism remains to be elucidated.

### Supplementary Information

Supplementary Information.

## Data Availability

Publicly available datasets were analyzed in this study. This data can be found here: The raw data and corresponding clinical information of TCGA were downloaded from the Genomic Data Commons (GDC, https://portal.gdc.cancer.gov/) and we filtered the patients with diagnosis of HNSC. In addition, raw data of GEO were downloaded from Gene Expression Omnibus Database (GEO; https://www.ncbi.nlm.nih.gov/geo/), especially GSE65858 (https://www.ncbi.nlm.nih.gov/geo/query/acc.cgi?acc=GSE658 58) and GSE31056 (https://www.ncbi.nlm.nih.gov/geo/query/acc.cgi?acc=GSE31056).

## Abbreviations

HNSC: Head and neck squamous cell carcinoma
LASSO: The Least Absolute Shrinkage and Selection Operator
OS: Overall survival
PFS: Progression-free survival
DEGs: Differentially expressed genes
OS: Overall survival
GO: Gene oncology
GSEA: Gene set enrichment analysis
HPV16: The Human papilloma virus 16
EGFR: Epidermal growth factor receptor
VEGF: Vascular endothelial growth factor
