# Supplementary material for: Identify and validate circadian regulators as potential prognostic markers and immune infiltrates in head and neck squamous cell carcinoma
Source: Sci Rep. 2023 Nov 15;13:19939. doi: 10.1038/s41598-023-46560-8 (PMC10651996; doi:10.1038/s41598-023-46560-8)
Supplement: Supplementary file 1 — Supplementary Information. [file 41598_2023_46560_MOESM1_ESM.docx]

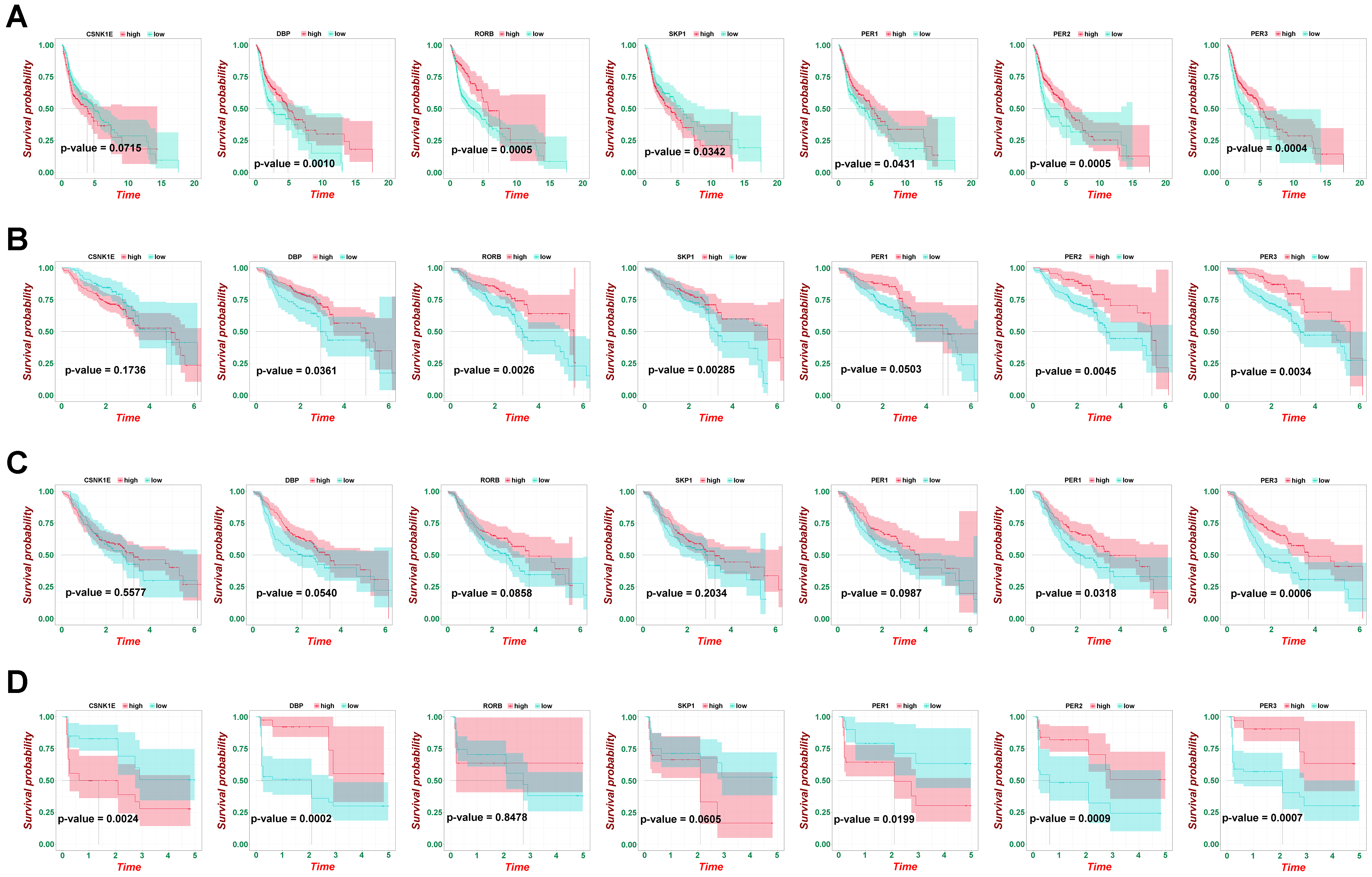


**Supplementary Figure 1.** Circadian hub gene selection. (**A**) The Kaplan–Meier plot of OS in TCGA based with 490 patients on 7 circadian genes. (**B**) The Kaplan–Meier plot of OS in GSE65858 with 270 patients based on 7 circadian genes. (**C**) The Kaplan–Meier plot of PFS in GSE65858 with 270 patients based on 7 circadian genes. (**D**) The Kaplan–Meier plot of PFS in GSE31056 with 96 patients based on 7 circadian genes.


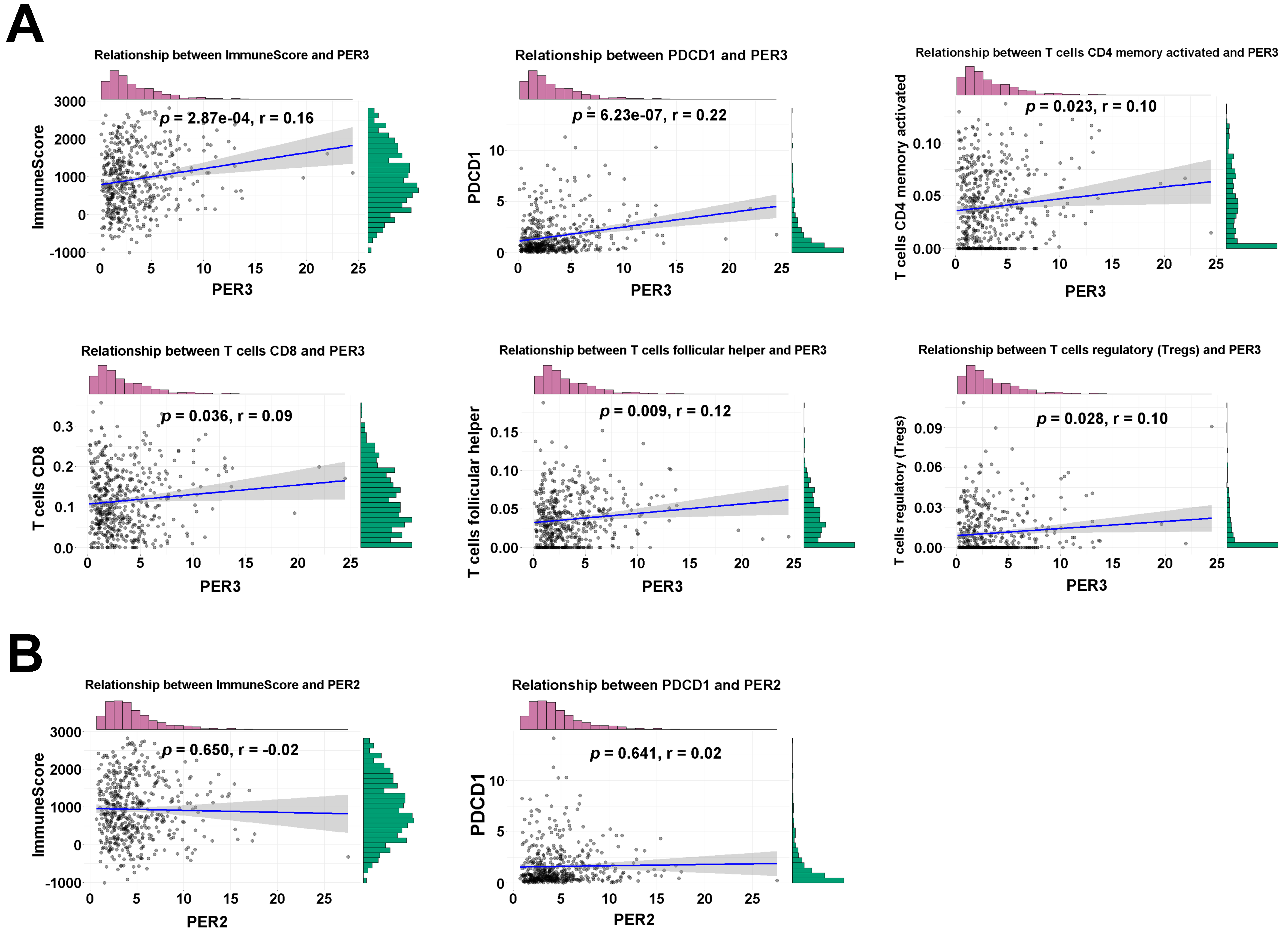


**Supplementary Figure 2.** The correlation between the PER2, PER3 and immune infiltration. (**A**) The correlation between PER3 and immune-related scores, biomarkers and immune cells. (**B**) The correlation between PER2 and immune-related scores, biomarkers and immune cells.

**
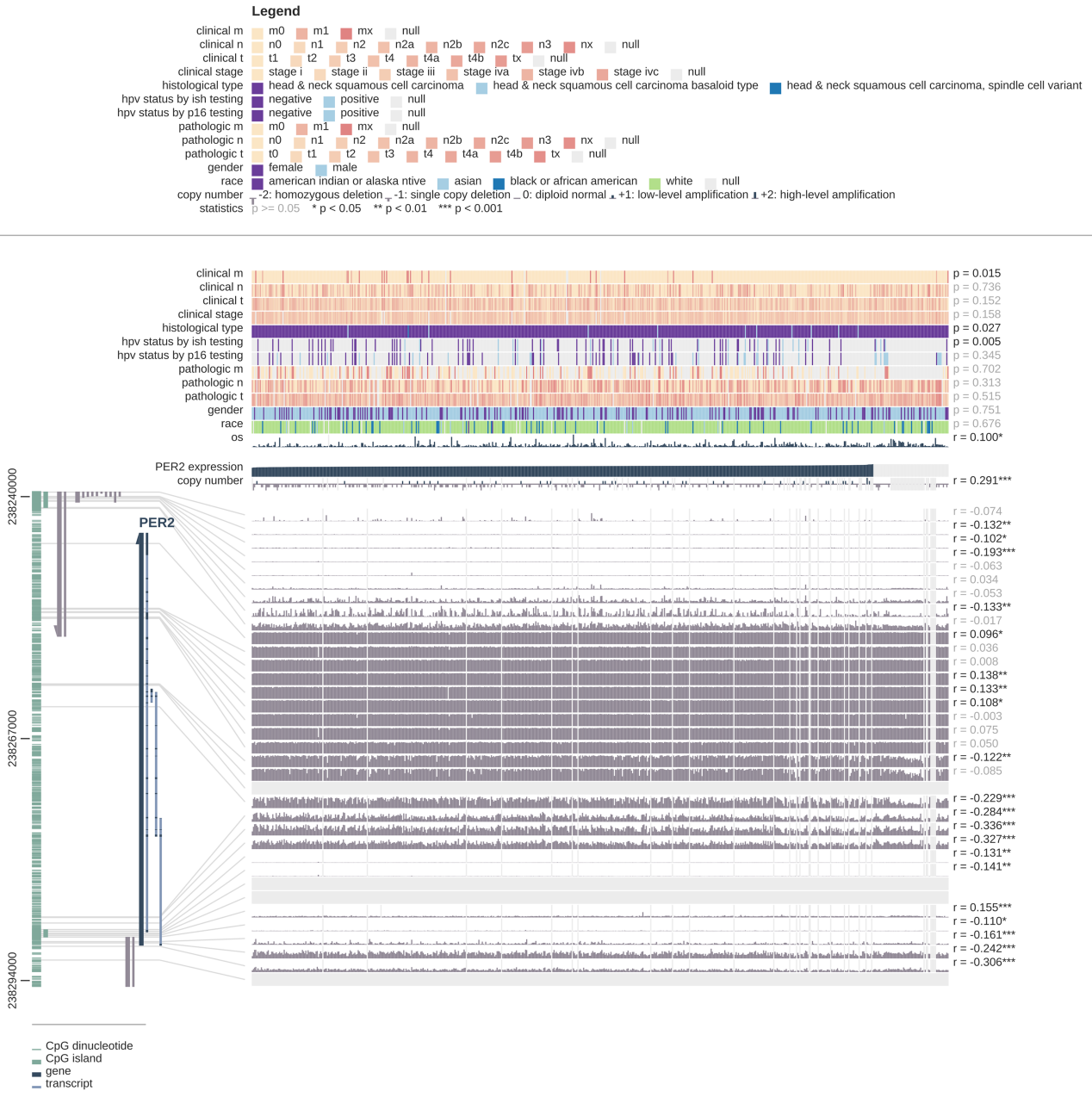
**

**Supplementary Figure 3.** The association between PER3 and clinical characteristics from MEXPRESS.

**
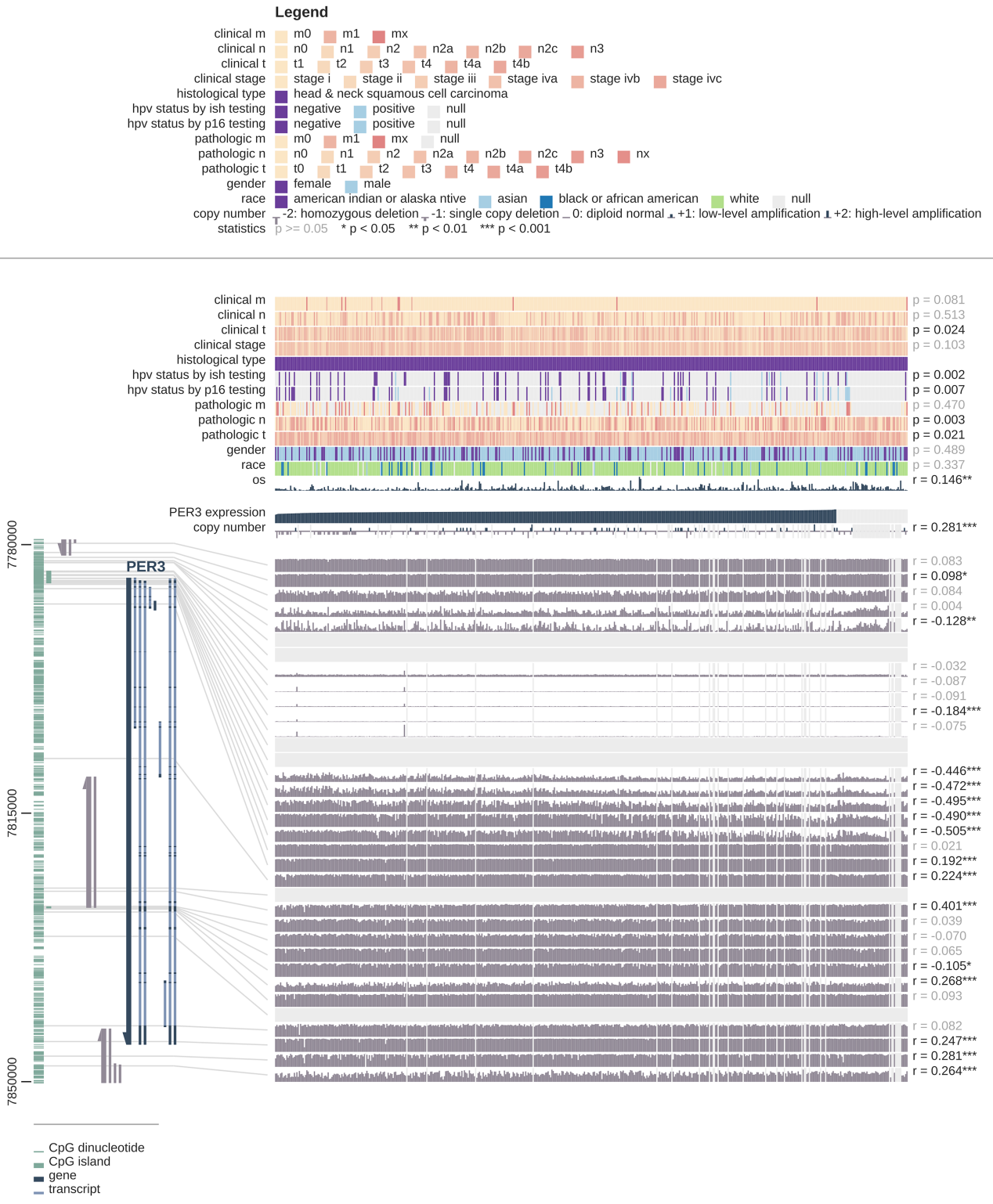
**

**Supplementary Figure 4.** The association between PER2 and clinical characteristics from MEXPRESS.


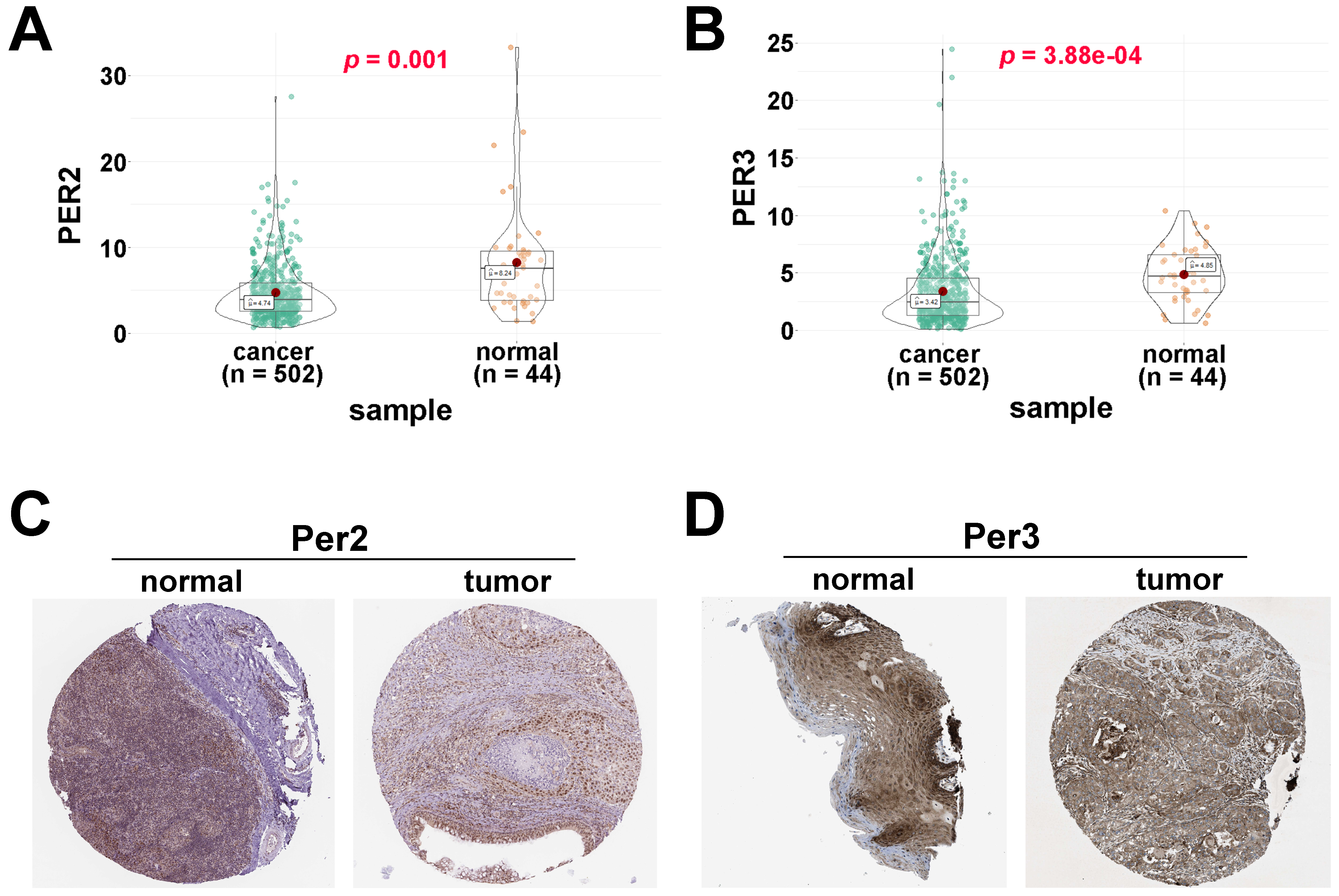


**Supplementary Figure 5.** The estimation of PER2, PER3 in normal and cancer tissue. (**A**) The expression of PER2 in normal and cancer tissue. (**B**) The expression of PER3 in normal and cancer tissue. (**C**) The immunohistochemistry of PER2 from HPA database. (**D**) The immunohistochemistry of PER3 from HPA database.

**Supplementary Table 1.** The Coefficient of 7 clock genes based on the risk model.

| **Gene** | **Regression coefficient** |
| --- | --- |
| CSNK1E | 0.000238057 |
| DBP | -0.032199989 |
| PER1 | -0.001559582 |
| PER2 | -0.01325351 |
| PER3 | -0.023593526 |
| RORB | -0.003212017 |
| SKP1 | 0.015601681 |

**Supplementary Table 2.** The sequences of primers and siRNAs used in the research.

| **Sequences of Primers** | |
| --- | --- |
| **Per2** | **Forward:** CTGGACTCCTCGGCTTGAAAC |
|  | **Reverse:** ATTCATGCTGGGCTCTGGAA |
| **Per3** | **Forward:** GCAGAGGAAATTGGCGGACA |
|  | **Reverse:** GGTTTATTGCGTCTCTCCGAG |
| **GAPDH** | **Forward:** GTCAGCCGCATCTT CTTT |
|  | **Reverse:** CGCCCAATACGACCAAAT |
| **Sequences of siRNAs** | |
| **si-Per2#1** | **Forward:** GGACAUGAGACCAACGAAATT |
|  | **Reverse:** UUUCGUUGGUCUCAUGUCCTT |
| **si-Per2#2** | **Forward:** CACCCAUACACCAAAUUGUTT |
|  | **Reverse:** ACAAUUUGGUGUAUGGGUGTT |
| **si-Per2#3** | **Forward:** GGCAGAAUGUGUUUACUGUTT |
|  | **Reverse:** ACAGUAAACACAUUCUGCCTT |
| **si-Per3#1** | **Forward:** GCCGGAAGAUUUCUUUCAUTT |
|  | **Reverse:** AUGAAAGAAAUCUUCCGGCTT |
| **si-Per3#2** | **Forward:** GACCUCGGAAGAAUUUAAATT |
|  | **Reverse:** UUUAAAUUCUUCCGAGGUCTT |
| **si-Per3#3** | **Forward:** GCUCACCCUUGCAGUUAAATT |
|  | **Reverse:** UUUAACUGCAAGGGUGAGCTT |
| **NC** | **Forward:** UUCUCCGAACGUGUCACGUTT |
|  | **Reverse:** ACGUGACACGUUCGGAGAATT |

**Supplementary Table 3.** The methylation level of PER3 from MEXPRESS

| # all comparisons were made against the region_expression data |  |  |
| --- | --- | --- |
| **Variable** | **p_value** | **pearson_r** |
| age_at_initial_pathologic_diagnosis | 0.00010388 | -0.182181525 |
| amount_of_alcohol_consumption_per_day | 0.983797317 | -0.001528646 |
| anatomic_neoplasm_subdivision | 4.29913E-05 | NA |
| anatomic_treatment_site | 0.691300563 | NA |
| clinical_m | 0.081108814 | NA |
| clinical_n | 0.512712936 | NA |
| clinical_t | 0.02374063 | NA |
| clinical_stage | 0.102923206 | NA |
| disease_after_curative_tx | 0.014823835 | NA |
| egfr_amplication_status | 0.826646872 | NA |
| frequency_of_alcohol_consumption | 0.866584132 | -0.012609273 |
| histological_type | NA | NA |
| history_of_neoadjuvant_treatment | 0.867994433 | NA |
| hpv_status_by_ish_testing | 0.002336404 | NA |
| hpv_status_by_p16_testing | 0.007117625 | NA |
| laterality | 0.236967875 | NA |
| lymphovascular_invasion_present | 0.317112305 | NA |
| margin_status | 0.116483339 | NA |
| neoplasm_histologic_grade | 0.644373388 | NA |
| new_neoplasm_event_occurrence_anatomic_site | 0.584722235 | NA |
| new_neoplasm_event_type | 1 | NA |
| new_tumor_event_after_initial_treatment | 0.139114701 | NA |
| number_of_lymphnodes_positive_by_he | 0.206528421 | -0.066000014 |
| number_of_lymphnodes_positive_by_ihc | 0.883482853 | 0.010077119 |
| number_pack_years_smoked | 0.917586687 | 0.00661742 |
| pathologic_m | 0.470404203 | NA |
| pathologic_n | 0.003420868 | NA |
| pathologic_t | 0.020804166 | NA |
| perineural_invasion_present | 0.428716552 | NA |
| person_neoplasm_cancer_status | 0.484501544 | NA |
| presence_of_pathological_nodal_extracapsular_spread | 0.79553036 | NA |
| primary_therapy_outcome_success | 0.181457804 | NA |
| smokeless_tobacco_use_per_day | NA | NA |
| smokeless_tobacco_use_regularly | 0.633104899 | NA |
| tissue_prospective_collection_indicator | 0.227017517 | NA |
| tissue_retrospective_collection_indicator | 0.227017517 | NA |
| tobacco_smoking_history | 0.917590996 | -0.004952397 |
| ethnicity | 0.169670664 | NA |
| gender | 0.489062036 | NA |
| race | 0.337099734 | NA |
| tumor_stage | 0.136739043 | NA |
| alcohol_history | 0.389733685 | NA |
| cigarettes_per_day | 0.917586687 | 0.00661742 |
| years_smoked | 0.358210138 | -0.084969477 |
| sample_type | 1.46E-09 | NA |
| tumor_stage_simplified | 0.135560579 | NA |
| clinical_stage_simplified | 0.137175141 | NA |
| os_event | 0.971572989 | 0.001686455 |
| os | 0.001958454 | 0.145758439 |
| cnv | 1.29E-08 | 0.280914621 |
| cg06722397 | 0.085902051 | 0.083211843 |
| cg13322290 | 0.043115298 | 0.0979323 |
| cg03263730 | 0.081501591 | 0.084400363 |
| cg22229039 | 0.939016182 | 0.003713295 |
| cg14511923 | 0.008274198 | -0.127643182 |
| cg02277850 | NA | NA |
| cg23987789 | NA | NA |
| cg11843502 | 0.514797885 | -0.031607529 |
| cg06487986 | 0.071317143 | -0.08736466 |
| cg23927002 | 0.059650559 | -0.091220242 |
| cg11753033 | 0.000133358 | -0.18386399 |
| cg17465881 | 0.122305119 | -0.074890614 |
| cg05803631 | NA | NA |
| cg01444397 | NA | NA |
| cg12258811 | 9.50E-20 | -0.446472429 |
| cg14204433 | 1.62E-21 | -0.471652824 |
| cg08764927 | 3.72E-23 | -0.494874522 |
| cg25514503 | 8.79E-23 | -0.489593311 |
| cg24454741 | 7.63E-24 | -0.504612115 |
| cg08134286 | 0.671544327 | 0.020578361 |
| cg09327610 | 6.42506E-05 | 0.192220875 |
| cg22507406 | 2.92239E-06 | 0.22432169 |
| cg10059324 | NA | NA |
| cg04837231 | 1.24E-16 | 0.401423844 |
| cg17328665 | 0.420522316 | 0.039081325 |
| cg04725166 | 0.146102689 | -0.070455866 |
| cg17724687 | 0.179406794 | 0.065094106 |
| cg08926642 | 0.030850357 | -0.104502047 |
| cg09168692 | 2.19E-08 | 0.26782309 |
| cg00019458 | 0.054045706 | 0.09329892 |
| cg11668188 | NA | NA |
| cg11978441 | 0.092491998 | 0.081520471 |
| cg00811891 | 2.57E-07 | 0.246821223 |
| cg14150148 | 4.39E-09 | 0.281105438 |
| cg00864860 | 3.54E-08 | 0.263828802 |

**Supplementary Table 4.** The methylation level of PER2 from MEXPRESS

| # all comparisons were made against the region_expression data |  |  |
| --- | --- | --- |
| **Variable** | **p_value** | **pearson_r** |
| age_at_initial_pathologic_diagnosis | 0.963754906 | -0.001951016 |
| amount_of_alcohol_consumption_per_day | 0.902765345 | 0.008208557 |
| anatomic_neoplasm_subdivision | 0.191807967 | NA |
| anatomic_treatment_site | 0.365554253 | NA |
| clinical_m | 0.014823351 | NA |
| clinical_n | 0.735595357 | NA |
| clinical_t | 0.152395896 | NA |
| clinical_stage | 0.157571018 | NA |
| disease_after_curative_tx | 0.065498407 | NA |
| egfr_amplication_status | 0.910710524 | NA |
| frequency_of_alcohol_consumption | 0.241641261 | 0.07785472 |
| histological_type | 0.026526818 | NA |
| history_of_neoadjuvant_treatment | 0.416362875 | NA |
| hpv_status_by_ish_testing | 0.004569635 | NA |
| hpv_status_by_p16_testing | 0.344955134 | NA |
| laterality | 0.114768383 | NA |
| lymphovascular_invasion_present | 0.912869504 | NA |
| margin_status | 0.533701092 | NA |
| neoplasm_histologic_grade | 0.574352125 | NA |
| new_neoplasm_event_occurrence_anatomic_site | 0.727699205 | NA |
| new_neoplasm_event_type | 0.068023966 | NA |
| new_tumor_event_after_initial_treatment | 0.060229958 | NA |
| number_of_lymphnodes_positive_by_he | 0.715142692 | -0.017859925 |
| number_of_lymphnodes_positive_by_ihc | 0.181086799 | -0.088885987 |
| number_pack_years_smoked | 0.303602541 | 0.058902575 |
| pathologic_m | 0.702131083 | NA |
| pathologic_n | 0.313408632 | NA |
| pathologic_t | 0.515195715 | NA |
| perineural_invasion_present | 0.199308586 | NA |
| person_neoplasm_cancer_status | 0.161222534 | NA |
| presence_of_pathological_nodal_extracapsular_spread | 0.047021743 | NA |
| primary_therapy_outcome_success | 0.123731758 | NA |
| smokeless_tobacco_use_per_day | NA | NA |
| smokeless_tobacco_use_regularly | 0.558263113 | NA |
| tissue_prospective_collection_indicator | 0.030191278 | NA |
| tissue_retrospective_collection_indicator | 0.030191278 | NA |
| tobacco_smoking_history | 0.237353633 | 0.051172428 |
| ethnicity | 0.648346946 | NA |
| gender | 0.750516955 | NA |
| race | 0.675513564 | NA |
| tumor_stage | 0.008938776 | NA |
| alcohol_history | 0.116822719 | NA |
| cigarettes_per_day | 0.303602541 | 0.058902575 |
| years_smoked | 0.597672638 | 0.043581543 |
| sample_type | 7.22E-14 | NA |
| tumor_stage_simplified | 0.00562372 | NA |
| clinical_stage_simplified | 0.147340922 | NA |
| os_event | 0.94424865 | -0.003002412 |
| os | 0.019007013 | 0.100442547 |
| cnv | 7.43E-11 | 0.290666051 |
| cg15319457 | 0.090786268 | -0.07410061 |
| cg17283117 | 0.002441062 | -0.132377879 |
| cg11027325 | 0.019665592 | -0.102076534 |
| cg09163117 | 8.94037E-06 | -0.193158649 |
| cg11815105 | 0.151103182 | -0.062925092 |
| cg10210789 | 0.436671271 | 0.034116444 |
| cg02105326 | 0.225700387 | -0.053116215 |
| cg24127874 | 0.002386336 | -0.13267406 |
| cg10976861 | 0.705791318 | -0.016561916 |
| cg06259818 | 0.027847652 | 0.096273843 |
| cg16914508 | 0.415511833 | 0.035712255 |
| cg25706474 | 0.8523446 | 0.008166035 |
| cg21315421 | 0.001584389 | 0.137926582 |
| cg21462970 | 0.002319278 | 0.133172662 |
| cg13787782 | 0.013667273 | 0.107873191 |
| cg04324746 | 0.949807037 | -0.002761837 |
| cg13469320 | 0.086332571 | 0.075140653 |
| cg20070418 | 0.254881747 | 0.049922556 |
| cg12308675 | 0.005265141 | -0.121966876 |
| cg22879834 | 0.051488523 | -0.085284176 |
| cg07719617 | NA | NA |
| cg24204951 | 1.30E-07 | -0.229154033 |
| cg23072629 | 6.07E-11 | -0.284397355 |
| cg11903188 | 1.67E-14 | -0.336265837 |
| cg08202720 | 8.20E-14 | -0.326544112 |
| cg23905308 | 0.002813938 | -0.130507487 |
| cg24831107 | 0.001224033 | -0.141146118 |
| cg16948082 | NA | NA |
| cg12912690 | NA | NA |
| cg02976543 | 0.000369054 | 0.155457037 |
| cg03004097 | 0.011733693 | -0.110228258 |
| cg05664072 | 0.000221356 | -0.160999266 |
| cg04169774 | 2.36E-08 | -0.242320301 |
| cg10780517 | 2.07E-12 | -0.306379147 |
| cg01011975 | NA | NA |

**Supplementary Table 5.** miRNAs combined with mRNAs in ceRNA.

| **miRNA** | **Gene** | **miRDB** | **miRTarBase** | **TargetScan** | **Sum** |
| --- | --- | --- | --- | --- | --- |
| hsa-mir-106a-5p | ARID4B | 1 | 1 | 1 | 3 |
| hsa-mir-519d-3p | ZNFX1 | 1 | 1 | 1 | 3 |
| hsa-mir-519d-3p | CENPQ | 1 | 1 | 1 | 3 |
| hsa-mir-106a-5p | MAPK1 | 1 | 1 | 1 | 3 |
| hsa-mir-137 | RORA | 1 | 1 | 1 | 3 |
| hsa-mir-519d-3p | PPP1R15B | 1 | 1 | 1 | 3 |
| hsa-mir-31-5p | MZT1 | 1 | 1 | 1 | 3 |
| hsa-mir-519d-3p | ANKRD50 | 1 | 1 | 1 | 3 |
| hsa-mir-106a-5p | RHOC | 1 | 1 | 1 | 3 |
| hsa-mir-137 | ESRRA | 1 | 1 | 1 | 3 |
| hsa-mir-106a-5p | MASTL | 1 | 1 | 1 | 3 |
| hsa-mir-106a-5p | RAD23B | 1 | 1 | 1 | 3 |
| hsa-mir-519d-3p | TNKS2 | 1 | 1 | 1 | 3 |
| hsa-mir-519d-3p | TNFRSF21 | 1 | 1 | 1 | 3 |
| hsa-mir-519d-3p | FBXL5 | 1 | 1 | 1 | 3 |
| hsa-mir-363-3p | GFPT2 | 1 | 1 | 1 | 3 |
| hsa-mir-519d-3p | REST | 1 | 1 | 1 | 3 |
| hsa-mir-519d-3p | NCOA3 | 1 | 1 | 1 | 3 |
| hsa-mir-363-3p | BCL2L11 | 1 | 1 | 1 | 3 |
| hsa-mir-106a-5p | SALL3 | 1 | 1 | 1 | 3 |
| hsa-mir-519d-3p | AGO1 | 1 | 1 | 1 | 3 |
| hsa-mir-519d-3p | ARHGAP35 | 1 | 1 | 1 | 3 |
| hsa-mir-519d-3p | CCND1 | 1 | 1 | 1 | 3 |
| hsa-mir-519d-3p | YOD1 | 1 | 1 | 1 | 3 |
| hsa-mir-106a-5p | ANKH | 1 | 1 | 1 | 3 |
| hsa-mir-519d-3p | POLR3G | 1 | 1 | 1 | 3 |
| hsa-mir-519d-3p | CNOT7 | 1 | 1 | 1 | 3 |
| hsa-mir-137 | GLIPR1 | 1 | 1 | 1 | 3 |
| hsa-mir-519d-3p | KIF23 | 1 | 1 | 1 | 3 |
| hsa-mir-106a-5p | MTF1 | 1 | 1 | 1 | 3 |
| hsa-mir-519d-3p | REEP3 | 1 | 1 | 1 | 3 |
| hsa-mir-106a-5p | CEP170 | 1 | 1 | 1 | 3 |
| hsa-mir-31-5p | KLF13 | 1 | 1 | 1 | 3 |
| hsa-mir-31-5p | ARF1 | 1 | 1 | 1 | 3 |
| hsa-mir-519d-3p | FBXO31 | 1 | 1 | 1 | 3 |
| hsa-mir-519d-3p | NHLRC3 | 1 | 1 | 1 | 3 |
| hsa-mir-137 | HNRNPDL | 1 | 1 | 1 | 3 |
| hsa-mir-519d-3p | BTG3 | 1 | 1 | 1 | 3 |
| hsa-mir-519d-3p | USP32 | 1 | 1 | 1 | 3 |
| hsa-mir-519d-3p | CLOCK | 1 | 1 | 1 | 3 |
| hsa-mir-106a-5p | KIAA0922 | 1 | 1 | 1 | 3 |
| hsa-mir-106a-5p | DYNC1LI2 | 1 | 1 | 1 | 3 |
| hsa-mir-519d-3p | DENND5B | 1 | 1 | 1 | 3 |
| hsa-mir-519d-3p | TRIM37 | 1 | 1 | 1 | 3 |
| hsa-mir-187-3p | DYRK2 | 1 | 1 | 1 | 3 |
| hsa-mir-106a-5p | RLIM | 1 | 1 | 1 | 3 |
| hsa-mir-519d-3p | HAUS8 | 1 | 1 | 1 | 3 |
| hsa-mir-137 | EOGT | 1 | 1 | 1 | 3 |
| hsa-mir-106a-5p | NABP1 | 1 | 1 | 1 | 3 |
| hsa-mir-137 | MITF | 1 | 1 | 1 | 3 |
| hsa-mir-519d-3p | CEP57 | 1 | 1 | 1 | 3 |
| hsa-mir-106a-5p | NPAT | 1 | 1 | 1 | 3 |
| hsa-mir-519d-3p | LASP1 | 1 | 1 | 1 | 3 |
| hsa-mir-519d-3p | KLHL20 | 1 | 1 | 1 | 3 |
| hsa-mir-106a-5p | RORA | 1 | 1 | 1 | 3 |
| hsa-mir-106a-5p | PTPDC1 | 1 | 1 | 1 | 3 |
| hsa-mir-363-3p | TBC1D8 | 1 | 1 | 1 | 3 |
| hsa-mir-519d-3p | CDKN1A | 1 | 1 | 1 | 3 |
| hsa-mir-106a-5p | SGTB | 1 | 1 | 1 | 3 |
| hsa-mir-363-3p | MAP2K4 | 1 | 1 | 1 | 3 |
| hsa-mir-106a-5p | SAMD12 | 1 | 1 | 1 | 3 |
| hsa-mir-137 | SFT2D3 | 1 | 1 | 1 | 3 |
| hsa-mir-137 | PAPD7 | 1 | 1 | 1 | 3 |
| hsa-mir-519d-3p | BTBD7 | 1 | 1 | 1 | 3 |
| hsa-mir-31-5p | NOL9 | 1 | 1 | 1 | 3 |
| hsa-mir-519d-3p | PPP1R3B | 1 | 1 | 1 | 3 |
| hsa-mir-519d-3p | GNS | 1 | 1 | 1 | 3 |
| hsa-mir-519d-3p | TMEM167A | 1 | 1 | 1 | 3 |
| hsa-mir-363-3p | PAPD7 | 1 | 1 | 1 | 3 |
| hsa-mir-519d-3p | DUSP2 | 1 | 1 | 1 | 3 |
| hsa-mir-137 | ZNF326 | 1 | 1 | 1 | 3 |
| hsa-mir-363-3p | ZFYVE21 | 1 | 1 | 1 | 3 |
| hsa-mir-106a-5p | TSG101 | 1 | 1 | 1 | 3 |
| hsa-mir-363-3p | FOXN2 | 1 | 1 | 1 | 3 |
| hsa-mir-363-3p | SLX4 | 1 | 1 | 1 | 3 |
| hsa-mir-519d-3p | DNAJC27 | 1 | 1 | 1 | 3 |
| hsa-mir-31-5p | FOXD4L5 | 1 | 1 | 1 | 3 |
| hsa-mir-106a-5p | RGMB | 1 | 1 | 1 | 3 |
| hsa-mir-206 | SMARCB1 | 1 | 1 | 1 | 3 |
| hsa-mir-106a-5p | SHOC2 | 1 | 1 | 1 | 3 |
| hsa-mir-106a-5p | CCDC71L | 1 | 1 | 1 | 3 |
| hsa-mir-519d-3p | RRAGD | 1 | 1 | 1 | 3 |
| hsa-mir-363-3p | ITPR1 | 1 | 1 | 1 | 3 |
| hsa-mir-106a-5p | LIMA1 | 1 | 1 | 1 | 3 |
| hsa-mir-106a-5p | EIF4G2 | 1 | 1 | 1 | 3 |
| hsa-mir-519d-3p | PPP3R1 | 1 | 1 | 1 | 3 |
| hsa-mir-363-3p | TRIM36 | 1 | 1 | 1 | 3 |
| hsa-mir-519d-3p | KPNA2 | 1 | 1 | 1 | 3 |
| hsa-mir-519d-3p | LYSMD3 | 1 | 1 | 1 | 3 |
| hsa-mir-519d-3p | PAFAH1B1 | 1 | 1 | 1 | 3 |
| hsa-mir-519d-3p | TET3 | 1 | 1 | 1 | 3 |
| hsa-mir-519d-3p | KIAA0513 | 1 | 1 | 1 | 3 |
| hsa-mir-519d-3p | LAPTM4A | 1 | 1 | 1 | 3 |
| hsa-mir-519d-3p | BNIP2 | 1 | 1 | 1 | 3 |
| hsa-mir-363-3p | S1PR1 | 1 | 1 | 1 | 3 |
| hsa-mir-137 | NCOA3 | 1 | 1 | 1 | 3 |
| hsa-mir-106a-5p | OSTM1 | 1 | 1 | 1 | 3 |
| hsa-mir-106a-5p | TXNIP | 1 | 1 | 1 | 3 |
| hsa-mir-106a-5p | U2SURP | 1 | 1 | 1 | 3 |
| hsa-mir-106a-5p | KLHL28 | 1 | 1 | 1 | 3 |
| hsa-mir-106a-5p | CAPN15 | 1 | 1 | 1 | 3 |
| hsa-mir-31-5p | PRKCE | 1 | 1 | 1 | 3 |
| hsa-mir-363-3p | TWF1 | 1 | 1 | 1 | 3 |
| hsa-mir-106a-5p | FCHO2 | 1 | 1 | 1 | 3 |
| hsa-mir-363-3p | PDPN | 1 | 1 | 1 | 3 |
| hsa-mir-106a-5p | MORF4L1 | 1 | 1 | 1 | 3 |
| hsa-mir-363-3p | GNAQ | 1 | 1 | 1 | 3 |
| hsa-mir-363-3p | CCDC113 | 1 | 1 | 1 | 3 |
| hsa-mir-106a-5p | ARHGAP1 | 1 | 1 | 1 | 3 |
| hsa-mir-106a-5p | DPYSL2 | 1 | 1 | 1 | 3 |
| hsa-mir-519d-3p | TP53INP1 | 1 | 1 | 1 | 3 |
| hsa-mir-363-3p | DUSP5 | 1 | 1 | 1 | 3 |
| hsa-mir-106a-5p | MAP3K2 | 1 | 1 | 1 | 3 |
| hsa-mir-519d-3p | TMEM123 | 1 | 1 | 1 | 3 |
| hsa-mir-519d-3p | TANC1 | 1 | 1 | 1 | 3 |
| hsa-mir-519d-3p | TMEM64 | 1 | 1 | 1 | 3 |
| hsa-mir-363-3p | YIPF4 | 1 | 1 | 1 | 3 |
| hsa-mir-519d-3p | CCSER2 | 1 | 1 | 1 | 3 |
| hsa-mir-106a-5p | LDLR | 1 | 1 | 1 | 3 |
| hsa-mir-106a-5p | ULK1 | 1 | 1 | 1 | 3 |
| hsa-mir-106a-5p | RPS6KA5 | 1 | 1 | 1 | 3 |
| hsa-mir-519d-3p | PIP4K2C | 1 | 1 | 1 | 3 |
| hsa-mir-519d-3p | RAP2C | 1 | 1 | 1 | 3 |
| hsa-mir-206 | MATR3 | 1 | 1 | 1 | 3 |
| hsa-mir-519d-3p | BRMS1L | 1 | 1 | 1 | 3 |
| hsa-mir-106a-5p | DNAJB9 | 1 | 1 | 1 | 3 |
| hsa-mir-519d-3p | E2F2 | 1 | 1 | 1 | 3 |
| hsa-mir-519d-3p | TNFAIP1 | 1 | 1 | 1 | 3 |
| hsa-mir-223-3p | HSP90B1 | 1 | 1 | 1 | 3 |
| hsa-mir-519d-3p | ZNF800 | 1 | 1 | 1 | 3 |
| hsa-mir-519d-3p | TGFBR2 | 1 | 1 | 1 | 3 |
| hsa-mir-519d-3p | FAM46C | 1 | 1 | 1 | 3 |
| hsa-mir-223-3p | SP3 | 1 | 1 | 1 | 3 |
| hsa-mir-206 | SFRP1 | 1 | 1 | 1 | 3 |
| hsa-mir-519d-3p | MKNK2 | 1 | 1 | 1 | 3 |
| hsa-mir-106a-5p | CRK | 1 | 1 | 1 | 3 |
| hsa-mir-106a-5p | CAPRIN2 | 1 | 1 | 1 | 3 |
| hsa-mir-519d-3p | ANKRD12 | 1 | 1 | 1 | 3 |
| hsa-mir-519d-3p | UBXN2A | 1 | 1 | 1 | 3 |
| hsa-mir-519d-3p | CHAF1A | 1 | 1 | 1 | 3 |
| hsa-mir-106a-5p | CNOT6L | 1 | 1 | 1 | 3 |
| hsa-mir-106a-5p | RAB11FIP1 | 1 | 1 | 1 | 3 |
| hsa-mir-519d-3p | FNBP1L | 1 | 1 | 1 | 3 |
| hsa-mir-519d-3p | VPS26A | 1 | 1 | 1 | 3 |
| hsa-mir-519d-3p | PTPN4 | 1 | 1 | 1 | 3 |
| hsa-mir-519d-3p | PITPNA | 1 | 1 | 1 | 3 |
| hsa-mir-106a-5p | HMGB3 | 1 | 1 | 1 | 3 |
| hsa-mir-106a-5p | GIGYF1 | 1 | 1 | 1 | 3 |
| hsa-mir-519d-3p | HAS2 | 1 | 1 | 1 | 3 |
| hsa-mir-363-3p | ZFC3H1 | 1 | 1 | 1 | 3 |
| hsa-mir-223-3p | MEF2C | 1 | 1 | 1 | 3 |
| hsa-mir-519d-3p | FYCO1 | 1 | 1 | 1 | 3 |
| hsa-mir-106a-5p | LIMK1 | 1 | 1 | 1 | 3 |
| hsa-mir-106a-5p | BICD2 | 1 | 1 | 1 | 3 |
| hsa-mir-31-5p | PPP2R2A | 1 | 1 | 1 | 3 |
| hsa-mir-363-3p | ERGIC2 | 1 | 1 | 1 | 3 |
| hsa-mir-106a-5p | PTGFRN | 1 | 1 | 1 | 3 |
| hsa-mir-223-3p | POLR3G | 1 | 1 | 1 | 3 |
| hsa-mir-206 | PAX3 | 1 | 1 | 1 | 3 |
| hsa-mir-519d-3p | EIF4H | 1 | 1 | 1 | 3 |
| hsa-mir-363-3p | GALNT7 | 1 | 1 | 1 | 3 |
| hsa-mir-519d-3p | PHTF2 | 1 | 1 | 1 | 3 |
| hsa-mir-137 | KIT | 1 | 1 | 1 | 3 |
| hsa-mir-363-3p | CNNM4 | 1 | 1 | 1 | 3 |
| hsa-mir-363-3p | ARID1B | 1 | 1 | 1 | 3 |
| hsa-mir-106a-5p | SUCO | 1 | 1 | 1 | 3 |
| hsa-mir-106a-5p | TMEM127 | 1 | 1 | 1 | 3 |
| hsa-mir-31-5p | NF2 | 1 | 1 | 1 | 3 |
| hsa-mir-106a-5p | PLS1 | 1 | 1 | 1 | 3 |
| hsa-mir-363-3p | PPP1R37 | 1 | 1 | 1 | 3 |
| hsa-mir-137 | GLO1 | 1 | 1 | 1 | 3 |
| hsa-mir-519d-3p | FBXO21 | 1 | 1 | 1 | 3 |
| hsa-mir-106a-5p | FEM1C | 1 | 1 | 1 | 3 |
| hsa-mir-106a-5p | SESN3 | 1 | 1 | 1 | 3 |
| hsa-mir-363-3p | SLC12A5 | 1 | 1 | 1 | 3 |
| hsa-mir-519d-3p | CLIP4 | 1 | 1 | 1 | 3 |
| hsa-mir-206 | NUP50 | 1 | 1 | 1 | 3 |
| hsa-mir-31-5p | FZD3 | 1 | 1 | 1 | 3 |
| hsa-mir-106a-5p | RAB10 | 1 | 1 | 1 | 3 |
| hsa-mir-106a-5p | GNB5 | 1 | 1 | 1 | 3 |
| hsa-mir-519d-3p | MIDN | 1 | 1 | 1 | 3 |
| hsa-mir-106a-5p | RUNX3 | 1 | 1 | 1 | 3 |
| hsa-mir-519d-3p | FAM129A | 1 | 1 | 1 | 3 |
| hsa-mir-106a-5p | ELK4 | 1 | 1 | 1 | 3 |
| hsa-mir-503-5p | RALGAPB | 1 | 1 | 1 | 3 |
| hsa-mir-137 | E2F6 | 1 | 1 | 1 | 3 |
| hsa-mir-519d-3p | SQSTM1 | 1 | 1 | 1 | 3 |
| hsa-mir-106a-5p | PPP6R3 | 1 | 1 | 1 | 3 |
| hsa-mir-519d-3p | EGLN3 | 1 | 1 | 1 | 3 |
| hsa-mir-106a-5p | RUFY2 | 1 | 1 | 1 | 3 |
| hsa-mir-31-5p | C19orf12 | 1 | 1 | 1 | 3 |
| hsa-mir-223-3p | LMO2 | 1 | 1 | 1 | 3 |
| hsa-mir-363-3p | MAP1B | 1 | 1 | 1 | 3 |
| hsa-mir-31-5p | PARP1 | 1 | 1 | 1 | 3 |
| hsa-mir-206 | ZNF215 | 1 | 1 | 1 | 3 |
| hsa-mir-519d-3p | UNK | 1 | 1 | 1 | 3 |
| hsa-mir-519d-3p | CERCAM | 1 | 1 | 1 | 3 |
| hsa-mir-519d-3p | SPRED1 | 1 | 1 | 1 | 3 |
| hsa-mir-106a-5p | MSMO1 | 1 | 1 | 1 | 3 |
| hsa-mir-519d-3p | PHF6 | 1 | 1 | 1 | 3 |
| hsa-mir-137 | LIMCH1 | 1 | 1 | 1 | 3 |
| hsa-mir-519d-3p | ZNF202 | 1 | 1 | 1 | 3 |
| hsa-mir-519d-3p | KLF3 | 1 | 1 | 1 | 3 |
| hsa-mir-106a-5p | ARHGAP12 | 1 | 1 | 1 | 3 |
| hsa-mir-106a-5p | SEMA7A | 1 | 1 | 1 | 3 |
| hsa-mir-519d-3p | NUP35 | 1 | 1 | 1 | 3 |
| hsa-mir-519d-3p | HBP1 | 1 | 1 | 1 | 3 |
| hsa-mir-519d-3p | ZBTB9 | 1 | 1 | 1 | 3 |
| hsa-mir-363-3p | RNF4 | 1 | 1 | 1 | 3 |
| hsa-mir-106a-5p | RRM2 | 1 | 1 | 1 | 3 |
| hsa-mir-106a-5p | RBL2 | 1 | 1 | 1 | 3 |
| hsa-mir-519d-3p | FAM126B | 1 | 1 | 1 | 3 |
| hsa-mir-223-3p | PTBP2 | 1 | 1 | 1 | 3 |
| hsa-mir-106a-5p | FAM210A | 1 | 1 | 1 | 3 |
| hsa-mir-137 | NCOA2 | 1 | 1 | 1 | 3 |
| hsa-mir-519d-3p | FRMD6 | 1 | 1 | 1 | 3 |
| hsa-mir-106a-5p | NKIRAS1 | 1 | 1 | 1 | 3 |
| hsa-mir-519d-3p | ZNF532 | 1 | 1 | 1 | 3 |
| hsa-mir-106a-5p | RAB22A | 1 | 1 | 1 | 3 |
| hsa-mir-106a-5p | SLC22A23 | 1 | 1 | 1 | 3 |
| hsa-mir-519d-3p | TWF1 | 1 | 1 | 1 | 3 |
| hsa-mir-519d-3p | ANKRD33B | 1 | 1 | 1 | 3 |
| hsa-mir-519d-3p | GBF1 | 1 | 1 | 1 | 3 |
| hsa-mir-206 | PGD | 1 | 1 | 1 | 3 |
| hsa-mir-519d-3p | FOXJ3 | 1 | 1 | 1 | 3 |
| hsa-mir-519d-3p | USP28 | 1 | 1 | 1 | 3 |
| hsa-mir-363-3p | TMF1 | 1 | 1 | 1 | 3 |
| hsa-mir-519d-3p | EEA1 | 1 | 1 | 1 | 3 |
| hsa-mir-519d-3p | PXK | 1 | 1 | 1 | 3 |
| hsa-mir-519d-3p | CHD9 | 1 | 1 | 1 | 3 |
| hsa-mir-519d-3p | 2-Sep | 1 | 1 | 1 | 3 |
| hsa-mir-519d-3p | PPP6C | 1 | 1 | 1 | 3 |
| hsa-mir-519d-3p | RAB5B | 1 | 1 | 1 | 3 |
| hsa-mir-106a-5p | EPHA4 | 1 | 1 | 1 | 3 |
| hsa-mir-106a-5p | ZC3H12C | 1 | 1 | 1 | 3 |
| hsa-mir-363-3p | ANP32E | 1 | 1 | 1 | 3 |
| hsa-mir-519d-3p | OCRL | 1 | 1 | 1 | 3 |
| hsa-mir-519d-3p | ANKRD52 | 1 | 1 | 1 | 3 |
| hsa-mir-106a-5p | MTMR3 | 1 | 1 | 1 | 3 |
| hsa-mir-519d-3p | SIK1 | 1 | 1 | 1 | 3 |
| hsa-mir-519d-3p | STAT3 | 1 | 1 | 1 | 3 |
| hsa-mir-519d-3p | SCAMP2 | 1 | 1 | 1 | 3 |
| hsa-mir-519d-3p | PFKP | 1 | 1 | 1 | 3 |
| hsa-mir-106a-5p | ZBTB7A | 1 | 1 | 1 | 3 |
| hsa-mir-519d-3p | BMPR2 | 1 | 1 | 1 | 3 |
| hsa-mir-106a-5p | KATNAL1 | 1 | 1 | 1 | 3 |
| hsa-mir-519d-3p | CHIC1 | 1 | 1 | 1 | 3 |
| hsa-mir-137 | SLC1A5 | 1 | 1 | 1 | 3 |
| hsa-mir-363-3p | GOLGA8B | 1 | 1 | 1 | 3 |
| hsa-mir-519d-3p | EZH1 | 1 | 1 | 1 | 3 |
| hsa-mir-106a-5p | ZBTB18 | 1 | 1 | 1 | 3 |
| hsa-mir-363-3p | HIVEP1 | 1 | 1 | 1 | 3 |
| hsa-mir-106a-5p | CEP97 | 1 | 1 | 1 | 3 |
| hsa-mir-519d-3p | NIN | 1 | 1 | 1 | 3 |
| hsa-mir-363-3p | MFF | 1 | 1 | 1 | 3 |
| hsa-mir-363-3p | REV3L | 1 | 1 | 1 | 3 |
| hsa-mir-363-3p | TOB1 | 1 | 1 | 1 | 3 |
| hsa-mir-503-5p | CREBL2 | 1 | 1 | 1 | 3 |
| hsa-mir-519d-3p | LPGAT1 | 1 | 1 | 1 | 3 |
| hsa-mir-106a-5p | CFL2 | 1 | 1 | 1 | 3 |
| hsa-mir-519d-3p | MAP3K3 | 1 | 1 | 1 | 3 |
| hsa-mir-223-3p | 2-Sep | 1 | 1 | 1 | 3 |
| hsa-mir-363-3p | KLHL15 | 1 | 1 | 1 | 3 |
| hsa-mir-519d-3p | SPOPL | 1 | 1 | 1 | 3 |
| hsa-mir-363-3p | INSIG1 | 1 | 1 | 1 | 3 |
| hsa-mir-519d-3p | TMBIM6 | 1 | 1 | 1 | 3 |
| hsa-mir-519d-3p | SOX4 | 1 | 1 | 1 | 3 |
| hsa-mir-519d-3p | ELAVL2 | 1 | 1 | 1 | 3 |
| hsa-mir-519d-3p | ACSL4 | 1 | 1 | 1 | 3 |
| hsa-mir-519d-3p | HMBOX1 | 1 | 1 | 1 | 3 |
| hsa-mir-106a-5p | PKNOX1 | 1 | 1 | 1 | 3 |
| hsa-mir-31-5p | CCNT1 | 1 | 1 | 1 | 3 |
| hsa-mir-31-5p | RHOBTB1 | 1 | 1 | 1 | 3 |
| hsa-mir-106a-5p | KIAA1147 | 1 | 1 | 1 | 3 |
| hsa-mir-519d-3p | SNTB2 | 1 | 1 | 1 | 3 |
| hsa-mir-106a-5p | HSPA8 | 1 | 1 | 1 | 3 |
| hsa-mir-31-5p | SP1 | 1 | 1 | 1 | 3 |
| hsa-mir-519d-3p | FJX1 | 1 | 1 | 1 | 3 |
| hsa-mir-106a-5p | EFCAB14 | 1 | 1 | 1 | 3 |
| hsa-mir-519d-3p | FRS2 | 1 | 1 | 1 | 3 |
| hsa-mir-106a-5p | F3 | 1 | 1 | 1 | 3 |
| hsa-mir-519d-3p | RBBP7 | 1 | 1 | 1 | 3 |
| hsa-mir-106a-5p | NETO2 | 1 | 1 | 1 | 3 |
| hsa-mir-363-3p | PCMTD1 | 1 | 1 | 1 | 3 |
| hsa-mir-363-3p | MYLIP | 1 | 1 | 1 | 3 |
| hsa-mir-106a-5p | GID4 | 1 | 1 | 1 | 3 |
| hsa-mir-106a-5p | NRIP3 | 1 | 1 | 1 | 3 |
| hsa-mir-519d-3p | ITCH | 1 | 1 | 1 | 3 |
| hsa-mir-519d-3p | USP3 | 1 | 1 | 1 | 3 |
| hsa-mir-106a-5p | NAGK | 1 | 1 | 1 | 3 |
| hsa-mir-519d-3p | WDR37 | 1 | 1 | 1 | 3 |
| hsa-mir-363-3p | VPS4B | 1 | 1 | 1 | 3 |
| hsa-mir-519d-3p | KLF10 | 1 | 1 | 1 | 3 |
| hsa-mir-206 | CERS2 | 1 | 1 | 1 | 3 |
| hsa-mir-363-3p | RRN3 | 1 | 1 | 1 | 3 |
| hsa-mir-206 | WEE1 | 1 | 1 | 1 | 3 |
| hsa-mir-106a-5p | SMOC1 | 1 | 1 | 1 | 3 |
| hsa-mir-519d-3p | ORMDL3 | 1 | 1 | 1 | 3 |
| hsa-mir-519d-3p | RAPGEF4 | 1 | 1 | 1 | 3 |
| hsa-mir-519d-3p | ZBTB4 | 1 | 1 | 1 | 3 |
| hsa-mir-519d-3p | TXLNA | 1 | 1 | 1 | 3 |
| hsa-mir-363-3p | GPBP1L1 | 1 | 1 | 1 | 3 |
| hsa-mir-519d-3p | CIT | 1 | 1 | 1 | 3 |
| hsa-mir-519d-3p | CREB1 | 1 | 1 | 1 | 3 |
| hsa-mir-363-3p | MYO1B | 1 | 1 | 1 | 3 |
| hsa-mir-503-5p | GREM2 | 1 | 1 | 1 | 3 |
| hsa-mir-223-3p | PRDM1 | 1 | 1 | 1 | 3 |
| hsa-mir-137 | PXN | 1 | 1 | 1 | 3 |
| hsa-mir-31-5p | FOXD4L4 | 1 | 1 | 1 | 3 |
| hsa-mir-363-3p | COX20 | 1 | 1 | 1 | 3 |
| hsa-mir-519d-3p | ZFYVE26 | 1 | 1 | 1 | 3 |
| hsa-mir-363-3p | UBXN4 | 1 | 1 | 1 | 3 |
| hsa-mir-106a-5p | WAC | 1 | 1 | 1 | 3 |
| hsa-mir-519d-3p | FOXQ1 | 1 | 1 | 1 | 3 |
| hsa-mir-363-3p | SERTAD3 | 1 | 1 | 1 | 3 |
| hsa-mir-31-5p | JAZF1 | 1 | 1 | 1 | 3 |
| hsa-mir-363-3p | PTAR1 | 1 | 1 | 1 | 3 |
| hsa-mir-106a-5p | ATL3 | 1 | 1 | 1 | 3 |
| hsa-mir-206 | HSP90B1 | 1 | 1 | 1 | 3 |
| hsa-mir-519d-3p | BBX | 1 | 1 | 1 | 3 |
| hsa-mir-519d-3p | PGM2L1 | 1 | 1 | 1 | 3 |
| hsa-mir-519d-3p | POLQ | 1 | 1 | 1 | 3 |
| hsa-mir-519d-3p | E2F5 | 1 | 1 | 1 | 3 |
| hsa-mir-519d-3p | RB1 | 1 | 1 | 1 | 3 |
| hsa-mir-519d-3p | PLEKHO2 | 1 | 1 | 1 | 3 |
| hsa-mir-137 | GIGYF1 | 1 | 1 | 1 | 3 |
| hsa-mir-31-5p | HIF1AN | 1 | 1 | 1 | 3 |
| hsa-mir-519d-3p | JAK1 | 1 | 1 | 1 | 3 |
| hsa-mir-363-3p | PAX9 | 1 | 1 | 1 | 3 |
| hsa-mir-519d-3p | NR2C2 | 1 | 1 | 1 | 3 |
| hsa-mir-106a-5p | CADM2 | 1 | 1 | 1 | 3 |
| hsa-mir-519d-3p | HIF1A | 1 | 1 | 1 | 3 |
| hsa-mir-363-3p | EXOC5 | 1 | 1 | 1 | 3 |
| hsa-mir-106a-5p | TRIP10 | 1 | 1 | 1 | 3 |
| hsa-mir-106a-5p | ATAD2 | 1 | 1 | 1 | 3 |
| hsa-mir-519d-3p | CYBRD1 | 1 | 1 | 1 | 3 |
| hsa-mir-31-5p | HOXC13 | 1 | 1 | 1 | 3 |
| hsa-mir-363-3p | SOX4 | 1 | 1 | 1 | 3 |
| hsa-mir-519d-3p | ZFYVE9 | 1 | 1 | 1 | 3 |
| hsa-mir-519d-3p | NFAT5 | 1 | 1 | 1 | 3 |
| hsa-mir-106a-5p | NACC2 | 1 | 1 | 1 | 3 |
| hsa-mir-519d-3p | E2F1 | 1 | 1 | 1 | 3 |
| hsa-mir-363-3p | CCSER2 | 1 | 1 | 1 | 3 |
| hsa-mir-206 | STC2 | 1 | 1 | 1 | 3 |
| hsa-mir-106a-5p | ENPP5 | 1 | 1 | 1 | 3 |
| hsa-mir-519d-3p | AKTIP | 1 | 1 | 1 | 3 |
| hsa-mir-363-3p | CPEB3 | 1 | 1 | 1 | 3 |
| hsa-mir-519d-3p | MINK1 | 1 | 1 | 1 | 3 |
| hsa-mir-519d-3p | MAPRE3 | 1 | 1 | 1 | 3 |
| hsa-mir-519d-3p | KMT2B | 1 | 1 | 1 | 3 |
| hsa-mir-519d-3p | FOXJ2 | 1 | 1 | 1 | 3 |
| hsa-mir-363-3p | KIF5B | 1 | 1 | 1 | 3 |
| hsa-mir-106a-5p | NRBP1 | 1 | 1 | 1 | 3 |
| hsa-mir-31-5p | ABCB9 | 1 | 1 | 1 | 3 |
| hsa-mir-206 | LRRC59 | 1 | 1 | 1 | 3 |
| hsa-mir-106a-5p | MCC | 1 | 1 | 1 | 3 |
| hsa-mir-363-3p | LHFPL2 | 1 | 1 | 1 | 3 |
| hsa-mir-106a-5p | TGFBR2 | 1 | 1 | 1 | 3 |
| hsa-mir-519d-3p | DNAJB9 | 1 | 1 | 1 | 3 |
| hsa-mir-363-3p | NUP43 | 1 | 1 | 1 | 3 |
| hsa-mir-106a-5p | UBXN2A | 1 | 1 | 1 | 3 |
| hsa-mir-519d-3p | PLXNA1 | 1 | 1 | 1 | 3 |
| hsa-mir-519d-3p | CRK | 1 | 1 | 1 | 3 |
| hsa-mir-519d-3p | CAPRIN2 | 1 | 1 | 1 | 3 |
| hsa-mir-106a-5p | MKNK2 | 1 | 1 | 1 | 3 |
| hsa-mir-519d-3p | ARAP2 | 1 | 1 | 1 | 3 |
| hsa-mir-206 | VAMP2 | 1 | 1 | 1 | 3 |
| hsa-mir-137 | CTBP1 | 1 | 1 | 1 | 3 |
| hsa-mir-106a-5p | TMEM64 | 1 | 1 | 1 | 3 |
| hsa-mir-519d-3p | RPS6KA5 | 1 | 1 | 1 | 3 |
| hsa-mir-363-3p | GOLGA3 | 1 | 1 | 1 | 3 |
| hsa-mir-106a-5p | RAP2C | 1 | 1 | 1 | 3 |
| hsa-mir-519d-3p | LDLR | 1 | 1 | 1 | 3 |
| hsa-mir-519d-3p | ULK1 | 1 | 1 | 1 | 3 |
| hsa-mir-519d-3p | SACS | 1 | 1 | 1 | 3 |
| hsa-mir-31-5p | STK40 | 1 | 1 | 1 | 3 |
| hsa-mir-223-3p | TWF1 | 1 | 1 | 1 | 3 |
| hsa-mir-137 | PTGS2 | 1 | 1 | 1 | 3 |
| hsa-mir-31-5p | ECHDC1 | 1 | 1 | 1 | 3 |
| hsa-mir-363-3p | SMU1 | 1 | 1 | 1 | 3 |
| hsa-mir-519d-3p | ARHGAP1 | 1 | 1 | 1 | 3 |
| hsa-mir-223-3p | CYB5A | 1 | 1 | 1 | 3 |
| hsa-mir-503-5p | CCND2 | 1 | 1 | 1 | 3 |
| hsa-mir-519d-3p | DDHD1 | 1 | 1 | 1 | 3 |
| hsa-mir-519d-3p | MAP3K2 | 1 | 1 | 1 | 3 |
| hsa-mir-106a-5p | TP53INP1 | 1 | 1 | 1 | 3 |
| hsa-mir-363-3p | ZDHHC5 | 1 | 1 | 1 | 3 |
| hsa-mir-223-3p | FOXO1 | 1 | 1 | 1 | 3 |
| hsa-mir-519d-3p | DPYSL2 | 1 | 1 | 1 | 3 |
| hsa-mir-519d-3p | CAPN15 | 1 | 1 | 1 | 3 |
| hsa-mir-31-5p | FOXD4 | 1 | 1 | 1 | 3 |
| hsa-mir-519d-3p | U2SURP | 1 | 1 | 1 | 3 |
| hsa-mir-519d-3p | KLHL28 | 1 | 1 | 1 | 3 |
| hsa-mir-519d-3p | TXNIP | 1 | 1 | 1 | 3 |
| hsa-mir-519d-3p | RPA2 | 1 | 1 | 1 | 3 |
| hsa-mir-519d-3p | MORF4L1 | 1 | 1 | 1 | 3 |
| hsa-mir-363-3p | SLC25A32 | 1 | 1 | 1 | 3 |
| hsa-mir-31-5p | SYDE2 | 1 | 1 | 1 | 3 |
| hsa-mir-519d-3p | FCHO2 | 1 | 1 | 1 | 3 |
| hsa-mir-519d-3p | ELK4 | 1 | 1 | 1 | 3 |
| hsa-mir-519d-3p | SSX2IP | 1 | 1 | 1 | 3 |
| hsa-mir-106a-5p | FAM129A | 1 | 1 | 1 | 3 |
| hsa-mir-519d-3p | RUNX3 | 1 | 1 | 1 | 3 |
| hsa-mir-106a-5p | MIDN | 1 | 1 | 1 | 3 |
| hsa-mir-206 | BSCL2 | 1 | 1 | 1 | 3 |
| hsa-mir-519d-3p | RUFY2 | 1 | 1 | 1 | 3 |
| hsa-mir-106a-5p | EGLN3 | 1 | 1 | 1 | 3 |
| hsa-mir-519d-3p | PPP6R3 | 1 | 1 | 1 | 3 |
| hsa-mir-363-3p | MED19 | 1 | 1 | 1 | 3 |
| hsa-mir-31-5p | GTF2E1 | 1 | 1 | 1 | 3 |
| hsa-mir-363-3p | GOLGA8J | 1 | 1 | 1 | 3 |
| hsa-mir-223-3p | IGF1R | 1 | 1 | 1 | 3 |
| hsa-mir-519d-3p | PLS1 | 1 | 1 | 1 | 3 |
| hsa-mir-519d-3p | TMEM127 | 1 | 1 | 1 | 3 |
| hsa-mir-519d-3p | RAB10 | 1 | 1 | 1 | 3 |
| hsa-mir-519d-3p | GNB5 | 1 | 1 | 1 | 3 |
| hsa-mir-363-3p | BTG2 | 1 | 1 | 1 | 3 |
| hsa-mir-519d-3p | CRY2 | 1 | 1 | 1 | 3 |
| hsa-mir-106a-5p | CLIP4 | 1 | 1 | 1 | 3 |
| hsa-mir-223-3p | FBXW7 | 1 | 1 | 1 | 3 |
| hsa-mir-519d-3p | ATG16L1 | 1 | 1 | 1 | 3 |
| hsa-mir-519d-3p | SESN3 | 1 | 1 | 1 | 3 |
| hsa-mir-519d-3p | FEM1C | 1 | 1 | 1 | 3 |
| hsa-mir-363-3p | BAK1 | 1 | 1 | 1 | 3 |
| hsa-mir-363-3p | TULP4 | 1 | 1 | 1 | 3 |
| hsa-mir-206 | G6PD | 1 | 1 | 1 | 3 |
| hsa-mir-519d-3p | BICD2 | 1 | 1 | 1 | 3 |
| hsa-mir-519d-3p | SUCO | 1 | 1 | 1 | 3 |
| hsa-mir-223-3p | ECT2 | 1 | 1 | 1 | 3 |
| hsa-mir-223-3p | F3 | 1 | 1 | 1 | 3 |
| hsa-mir-106a-5p | PHTF2 | 1 | 1 | 1 | 3 |
| hsa-mir-106a-5p | EIF4H | 1 | 1 | 1 | 3 |
| hsa-mir-519d-3p | PTGFRN | 1 | 1 | 1 | 3 |
| hsa-mir-106a-5p | PTPN4 | 1 | 1 | 1 | 3 |
| hsa-mir-519d-3p | C7orf43 | 1 | 1 | 1 | 3 |
| hsa-mir-363-3p | CIC | 1 | 1 | 1 | 3 |
| hsa-mir-206 | RNF138 | 1 | 1 | 1 | 3 |
| hsa-mir-106a-5p | FNBP1L | 1 | 1 | 1 | 3 |
| hsa-mir-519d-3p | CNOT6L | 1 | 1 | 1 | 3 |
| hsa-mir-206 | KRAS | 1 | 1 | 1 | 3 |
| hsa-mir-519d-3p | GIGYF1 | 1 | 1 | 1 | 3 |
| hsa-mir-106a-5p | HAS2 | 1 | 1 | 1 | 3 |
| hsa-mir-31-5p | RASA1 | 1 | 1 | 1 | 3 |
| hsa-mir-106a-5p | PITPNA | 1 | 1 | 1 | 3 |
| hsa-mir-519d-3p | HMGB3 | 1 | 1 | 1 | 3 |
| hsa-mir-106a-5p | TRIM37 | 1 | 1 | 1 | 3 |
| hsa-mir-519d-3p | DYNC1LI2 | 1 | 1 | 1 | 3 |
| hsa-mir-206 | EIF1AX | 1 | 1 | 1 | 3 |
| hsa-mir-519d-3p | AKT3 | 1 | 1 | 1 | 3 |
| hsa-mir-519d-3p | RAB11FIP1 | 1 | 1 | 1 | 3 |
| hsa-mir-519d-3p | KIAA0922 | 1 | 1 | 1 | 3 |
| hsa-mir-519d-3p | GOLGA1 | 1 | 1 | 1 | 3 |
| hsa-mir-519d-3p | SLC16A9 | 1 | 1 | 1 | 3 |
| hsa-mir-519d-3p | NPAT | 1 | 1 | 1 | 3 |
| hsa-mir-206 | TKT | 1 | 1 | 1 | 3 |
| hsa-mir-31-5p | SELE | 1 | 1 | 1 | 3 |
| hsa-mir-519d-3p | NABP1 | 1 | 1 | 1 | 3 |
| hsa-mir-363-3p | TPPP | 1 | 1 | 1 | 3 |
| hsa-mir-106a-5p | HAUS8 | 1 | 1 | 1 | 3 |
| hsa-mir-519d-3p | RLIM | 1 | 1 | 1 | 3 |
| hsa-mir-106a-5p | KIF23 | 1 | 1 | 1 | 3 |
| hsa-mir-31-5p | YWHAE | 1 | 1 | 1 | 3 |
| hsa-mir-519d-3p | ANKH | 1 | 1 | 1 | 3 |
| hsa-mir-519d-3p | SCAMP5 | 1 | 1 | 1 | 3 |
| hsa-mir-106a-5p | YOD1 | 1 | 1 | 1 | 3 |
| hsa-mir-519d-3p | SLK | 1 | 1 | 1 | 3 |
| hsa-mir-106a-5p | USP32 | 1 | 1 | 1 | 3 |
| hsa-mir-137 | YBX1 | 1 | 1 | 1 | 3 |
| hsa-mir-106a-5p | BTG3 | 1 | 1 | 1 | 3 |
| hsa-mir-519d-3p | SEMA4B | 1 | 1 | 1 | 3 |
| hsa-mir-223-3p | FOXO3 | 1 | 1 | 1 | 3 |
| hsa-mir-519d-3p | CEP170 | 1 | 1 | 1 | 3 |
| hsa-mir-519d-3p | MTF1 | 1 | 1 | 1 | 3 |
| hsa-mir-106a-5p | REEP3 | 1 | 1 | 1 | 3 |
| hsa-mir-363-3p | BAZ2B | 1 | 1 | 1 | 3 |
| hsa-mir-363-3p | SOX11 | 1 | 1 | 1 | 3 |
| hsa-mir-106a-5p | FBXL5 | 1 | 1 | 1 | 3 |
| hsa-mir-31-5p | ARID1A | 1 | 1 | 1 | 3 |
| hsa-mir-106a-5p | CCND1 | 1 | 1 | 1 | 3 |
| hsa-mir-206 | GJA1 | 1 | 1 | 1 | 3 |
| hsa-mir-106a-5p | AGO1 | 1 | 1 | 1 | 3 |
| hsa-mir-137 | DR1 | 1 | 1 | 1 | 3 |
| hsa-mir-519d-3p | SALL3 | 1 | 1 | 1 | 3 |
| hsa-mir-106a-5p | REST | 1 | 1 | 1 | 3 |
| hsa-mir-519d-3p | TOPORS | 1 | 1 | 1 | 3 |
| hsa-mir-363-3p | KIAA1109 | 1 | 1 | 1 | 3 |
| hsa-mir-106a-5p | ANKRD50 | 1 | 1 | 1 | 3 |
| hsa-mir-519d-3p | RHOC | 1 | 1 | 1 | 3 |
| hsa-mir-106a-5p | PPP1R15B | 1 | 1 | 1 | 3 |
| hsa-mir-519d-3p | MAPK1 | 1 | 1 | 1 | 3 |
| hsa-mir-106a-5p | ZNFX1 | 1 | 1 | 1 | 3 |
| hsa-mir-106a-5p | CENPQ | 1 | 1 | 1 | 3 |
| hsa-mir-519d-3p | ARID4B | 1 | 1 | 1 | 3 |
| hsa-mir-106a-5p | TNFRSF21 | 1 | 1 | 1 | 3 |
| hsa-mir-106a-5p | TNKS2 | 1 | 1 | 1 | 3 |
| hsa-mir-363-3p | NUFIP2 | 1 | 1 | 1 | 3 |
| hsa-mir-519d-3p | MASTL | 1 | 1 | 1 | 3 |
| hsa-mir-106a-5p | LAPTM4A | 1 | 1 | 1 | 3 |
| hsa-mir-106a-5p | KIAA0513 | 1 | 1 | 1 | 3 |
| hsa-mir-519d-3p | TADA2B | 1 | 1 | 1 | 3 |
| hsa-mir-206 | FRS2 | 1 | 1 | 1 | 3 |
| hsa-mir-106a-5p | KPNA2 | 1 | 1 | 1 | 3 |
| hsa-mir-363-3p | GTF2A1 | 1 | 1 | 1 | 3 |
| hsa-mir-519d-3p | OSTM1 | 1 | 1 | 1 | 3 |
| hsa-mir-106a-5p | BNIP2 | 1 | 1 | 1 | 3 |
| hsa-mir-519d-3p | PARD6B | 1 | 1 | 1 | 3 |
| hsa-mir-519d-3p | CCDC71L | 1 | 1 | 1 | 3 |
| hsa-mir-519d-3p | SHOC2 | 1 | 1 | 1 | 3 |
| hsa-mir-519d-3p | RGMB | 1 | 1 | 1 | 3 |
| hsa-mir-363-3p | C11orf24 | 1 | 1 | 1 | 3 |
| hsa-mir-137 | CSE1L | 1 | 1 | 1 | 3 |
| hsa-mir-519d-3p | LIMA1 | 1 | 1 | 1 | 3 |
| hsa-mir-363-3p | VMA21 | 1 | 1 | 1 | 3 |
| hsa-mir-223-3p | CFTR | 1 | 1 | 1 | 3 |
| hsa-mir-106a-5p | RRAGD | 1 | 1 | 1 | 3 |
| hsa-mir-223-3p | CDC27 | 1 | 1 | 1 | 3 |
| hsa-mir-31-5p | LATS2 | 1 | 1 | 1 | 3 |
| hsa-mir-519d-3p | SAMD12 | 1 | 1 | 1 | 3 |
| hsa-mir-519d-3p | TSG101 | 1 | 1 | 1 | 3 |
| hsa-mir-363-3p | MCOLN2 | 1 | 1 | 1 | 3 |
| hsa-mir-223-3p | RIF1 | 1 | 1 | 1 | 3 |
| hsa-mir-363-3p | PHLPP2 | 1 | 1 | 1 | 3 |
| hsa-mir-106a-5p | DUSP2 | 1 | 1 | 1 | 3 |
| hsa-mir-106a-5p | GNS | 1 | 1 | 1 | 3 |
| hsa-mir-519d-3p | MCL1 | 1 | 1 | 1 | 3 |
| hsa-mir-519d-3p | FAM102A | 1 | 1 | 1 | 3 |
| hsa-mir-106a-5p | LASP1 | 1 | 1 | 1 | 3 |
| hsa-mir-519d-3p | DDX5 | 1 | 1 | 1 | 3 |
| hsa-mir-519d-3p | SGTB | 1 | 1 | 1 | 3 |
| hsa-mir-106a-5p | CDKN1A | 1 | 1 | 1 | 3 |
| hsa-mir-519d-3p | RORA | 1 | 1 | 1 | 3 |
| hsa-mir-519d-3p | PTPDC1 | 1 | 1 | 1 | 3 |
| hsa-mir-519d-3p | WAC | 1 | 1 | 1 | 3 |
| hsa-mir-519d-3p | UBE2Q2 | 1 | 1 | 1 | 3 |
| hsa-mir-519d-3p | ABCA1 | 1 | 1 | 1 | 3 |
| hsa-mir-106a-5p | ZFYVE26 | 1 | 1 | 1 | 3 |
| hsa-mir-31-5p | FOXD4L1 | 1 | 1 | 1 | 3 |
| hsa-mir-503-5p | ZNF282 | 1 | 1 | 1 | 3 |
| hsa-mir-519d-3p | MLLT1 | 1 | 1 | 1 | 3 |
| hsa-mir-519d-3p | PANK3 | 1 | 1 | 1 | 3 |
| hsa-mir-363-3p | CNEP1R1 | 1 | 1 | 1 | 3 |
| hsa-mir-106a-5p | FOXQ1 | 1 | 1 | 1 | 3 |
| hsa-mir-31-5p | TBXA2R | 1 | 1 | 1 | 3 |
| hsa-mir-137 | SNRK | 1 | 1 | 1 | 3 |
| hsa-mir-137 | AGO4 | 1 | 1 | 1 | 3 |
| hsa-mir-106a-5p | CREB1 | 1 | 1 | 1 | 3 |
| hsa-mir-519d-3p | SMOC1 | 1 | 1 | 1 | 3 |
| hsa-mir-137 | YTHDF3 | 1 | 1 | 1 | 3 |
| hsa-mir-363-3p | GOLGA8A | 1 | 1 | 1 | 3 |
| hsa-mir-106a-5p | ZBTB4 | 1 | 1 | 1 | 3 |
| hsa-mir-519d-3p | STK17B | 1 | 1 | 1 | 3 |
| hsa-mir-223-3p | ZNF365 | 1 | 1 | 1 | 3 |
| hsa-mir-519d-3p | RUNDC1 | 1 | 1 | 1 | 3 |
| hsa-mir-363-3p | BCL11B | 1 | 1 | 1 | 3 |
| hsa-mir-519d-3p | NRIP3 | 1 | 1 | 1 | 3 |
| hsa-mir-363-3p | SLC25A36 | 1 | 1 | 1 | 3 |
| hsa-mir-519d-3p | GID4 | 1 | 1 | 1 | 3 |
| hsa-mir-519d-3p | NETO2 | 1 | 1 | 1 | 3 |
| hsa-mir-106a-5p | FRS2 | 1 | 1 | 1 | 3 |
| hsa-mir-519d-3p | F3 | 1 | 1 | 1 | 3 |
| hsa-mir-363-3p | USP28 | 1 | 1 | 1 | 3 |
| hsa-mir-519d-3p | UXS1 | 1 | 1 | 1 | 3 |
| hsa-mir-106a-5p | KLF10 | 1 | 1 | 1 | 3 |
| hsa-mir-206 | KCNJ2 | 1 | 1 | 1 | 3 |
| hsa-mir-519d-3p | NAGK | 1 | 1 | 1 | 3 |
| hsa-mir-31-5p | NUMB | 1 | 1 | 1 | 3 |
| hsa-mir-137 | RREB1 | 1 | 1 | 1 | 3 |
| hsa-mir-519d-3p | NRBP1 | 1 | 1 | 1 | 3 |
| hsa-mir-503-5p | CCND1 | 1 | 1 | 1 | 3 |
| hsa-mir-519d-3p | ABHD2 | 1 | 1 | 1 | 3 |
| hsa-mir-363-3p | DDX3X | 1 | 1 | 1 | 3 |
| hsa-mir-206 | ANP32B | 1 | 1 | 1 | 3 |
| hsa-mir-519d-3p | TMEM245 | 1 | 1 | 1 | 3 |
| hsa-mir-519d-3p | STX6 | 1 | 1 | 1 | 3 |
| hsa-mir-519d-3p | MCC | 1 | 1 | 1 | 3 |
| hsa-mir-363-3p | SSFA2 | 1 | 1 | 1 | 3 |
| hsa-mir-519d-3p | PIP4K2A | 1 | 1 | 1 | 3 |
| hsa-mir-363-3p | MOAP1 | 1 | 1 | 1 | 3 |
| hsa-mir-31-5p | ZNF805 | 1 | 1 | 1 | 3 |
| hsa-mir-31-5p | ZC3H12C | 1 | 1 | 1 | 3 |
| hsa-mir-519d-3p | ENPP5 | 1 | 1 | 1 | 3 |
| hsa-mir-519d-3p | PTEN | 1 | 1 | 1 | 3 |
| hsa-mir-223-3p | NFIA | 1 | 1 | 1 | 3 |
| hsa-mir-519d-3p | RABEP1 | 1 | 1 | 1 | 3 |
| hsa-mir-106a-5p | FOXJ2 | 1 | 1 | 1 | 3 |
| hsa-mir-106a-5p | KMT2B | 1 | 1 | 1 | 3 |
| hsa-mir-106a-5p | MAPRE3 | 1 | 1 | 1 | 3 |
| hsa-mir-106a-5p | MINK1 | 1 | 1 | 1 | 3 |
| hsa-mir-519d-3p | ATAD2 | 1 | 1 | 1 | 3 |
| hsa-mir-519d-3p | TRIP10 | 1 | 1 | 1 | 3 |
| hsa-mir-106a-5p | NR2C2 | 1 | 1 | 1 | 3 |
| hsa-mir-223-3p | PAX6 | 1 | 1 | 1 | 3 |
| hsa-mir-363-3p | SPRYD4 | 1 | 1 | 1 | 3 |
| hsa-mir-519d-3p | NACC2 | 1 | 1 | 1 | 3 |
| hsa-mir-106a-5p | E2F1 | 1 | 1 | 1 | 3 |
| hsa-mir-106a-5p | NFAT5 | 1 | 1 | 1 | 3 |
| hsa-mir-106a-5p | ZFYVE9 | 1 | 1 | 1 | 3 |
| hsa-mir-363-3p | SESN3 | 1 | 1 | 1 | 3 |
| hsa-mir-106a-5p | ZBTB47 | 1 | 1 | 1 | 3 |
| hsa-mir-206 | FNDC3A | 1 | 1 | 1 | 3 |
| hsa-mir-363-3p | FNIP1 | 1 | 1 | 1 | 3 |
| hsa-mir-519d-3p | VPS13C | 1 | 1 | 1 | 3 |
| hsa-mir-519d-3p | ATL3 | 1 | 1 | 1 | 3 |
| hsa-mir-106a-5p | BBX | 1 | 1 | 1 | 3 |
| hsa-mir-363-3p | PLEKHA1 | 1 | 1 | 1 | 3 |
| hsa-mir-519d-3p | ANKIB1 | 1 | 1 | 1 | 3 |
| hsa-mir-106a-5p | RB1 | 1 | 1 | 1 | 3 |
| hsa-mir-223-3p | RRAS2 | 1 | 1 | 1 | 3 |
| hsa-mir-363-3p | KLHDC10 | 1 | 1 | 1 | 3 |
| hsa-mir-363-3p | UBE2Z | 1 | 1 | 1 | 3 |
| hsa-mir-519d-3p | ATG14 | 1 | 1 | 1 | 3 |
| hsa-mir-106a-5p | PXK | 1 | 1 | 1 | 3 |
| hsa-mir-137 | FMNL2 | 1 | 1 | 1 | 3 |
| hsa-mir-363-3p | GID4 | 1 | 1 | 1 | 3 |
| hsa-mir-519d-3p | EPHA4 | 1 | 1 | 1 | 3 |
| hsa-mir-363-3p | GEMIN2 | 1 | 1 | 1 | 3 |
| hsa-mir-363-3p | TEF | 1 | 1 | 1 | 3 |
| hsa-mir-106a-5p | RAB5B | 1 | 1 | 1 | 3 |
| hsa-mir-106a-5p | 2-Sep | 1 | 1 | 1 | 3 |
| hsa-mir-363-3p | FASLG | 1 | 1 | 1 | 3 |
| hsa-mir-519d-3p | NKIRAS1 | 1 | 1 | 1 | 3 |
| hsa-mir-137 | KDM1A | 1 | 1 | 1 | 3 |
| hsa-mir-519d-3p | GAB1 | 1 | 1 | 1 | 3 |
| hsa-mir-519d-3p | OXR1 | 1 | 1 | 1 | 3 |
| hsa-mir-106a-5p | FOXJ3 | 1 | 1 | 1 | 3 |
| hsa-mir-223-3p | SMARCD1 | 1 | 1 | 1 | 3 |
| hsa-mir-519d-3p | SLAIN2 | 1 | 1 | 1 | 3 |
| hsa-mir-223-3p | EPB41L3 | 1 | 1 | 1 | 3 |
| hsa-mir-106a-5p | ANKRD33B | 1 | 1 | 1 | 3 |
| hsa-mir-503-5p | ZNRF2 | 1 | 1 | 1 | 3 |
| hsa-mir-363-3p | RSBN1 | 1 | 1 | 1 | 3 |
| hsa-mir-106a-5p | ZNF532 | 1 | 1 | 1 | 3 |
| hsa-mir-519d-3p | RAB22A | 1 | 1 | 1 | 3 |
| hsa-mir-519d-3p | SLC22A23 | 1 | 1 | 1 | 3 |
| hsa-mir-519d-3p | RRM2 | 1 | 1 | 1 | 3 |
| hsa-mir-519d-3p | CMPK1 | 1 | 1 | 1 | 3 |
| hsa-mir-519d-3p | ANKRD13C | 1 | 1 | 1 | 3 |
| hsa-mir-519d-3p | FBXO48 | 1 | 1 | 1 | 3 |
| hsa-mir-519d-3p | FAM210A | 1 | 1 | 1 | 3 |
| hsa-mir-106a-5p | FAM126B | 1 | 1 | 1 | 3 |
| hsa-mir-206 | BDNF | 1 | 1 | 1 | 3 |
| hsa-mir-223-3p | PHF19 | 1 | 1 | 1 | 3 |
| hsa-mir-106a-5p | SPRED1 | 1 | 1 | 1 | 3 |
| hsa-mir-106a-5p | CERCAM | 1 | 1 | 1 | 3 |
| hsa-mir-106a-5p | UNK | 1 | 1 | 1 | 3 |
| hsa-mir-106a-5p | HBP1 | 1 | 1 | 1 | 3 |
| hsa-mir-519d-3p | SEMA7A | 1 | 1 | 1 | 3 |
| hsa-mir-519d-3p | ARHGAP12 | 1 | 1 | 1 | 3 |
| hsa-mir-106a-5p | ZNF202 | 1 | 1 | 1 | 3 |
| hsa-mir-106a-5p | KLF3 | 1 | 1 | 1 | 3 |
| hsa-mir-363-3p | DNAJB9 | 1 | 1 | 1 | 3 |
| hsa-mir-519d-3p | KIAA1147 | 1 | 1 | 1 | 3 |
| hsa-mir-519d-3p | C14orf28 | 1 | 1 | 1 | 3 |
| hsa-mir-223-3p | TOX | 1 | 1 | 1 | 3 |
| hsa-mir-206 | GPD2 | 1 | 1 | 1 | 3 |
| hsa-mir-363-3p | EDEM1 | 1 | 1 | 1 | 3 |
| hsa-mir-519d-3p | PKNOX1 | 1 | 1 | 1 | 3 |
| hsa-mir-106a-5p | HMBOX1 | 1 | 1 | 1 | 3 |
| hsa-mir-519d-3p | PRR14L | 1 | 1 | 1 | 3 |
| hsa-mir-106a-5p | FJX1 | 1 | 1 | 1 | 3 |
| hsa-mir-519d-3p | EFCAB14 | 1 | 1 | 1 | 3 |
| hsa-mir-223-3p | FAM60A | 1 | 1 | 1 | 3 |
| hsa-mir-519d-3p | HSPA8 | 1 | 1 | 1 | 3 |
| hsa-mir-363-3p | CNIH1 | 1 | 1 | 1 | 3 |
| hsa-mir-503-5p | JARID2 | 1 | 1 | 1 | 3 |
| hsa-mir-363-3p | PER2 | 1 | 1 | 1 | 3 |
| hsa-mir-519d-3p | EIF5A2 | 1 | 1 | 1 | 3 |
| hsa-mir-519d-3p | CFL2 | 1 | 1 | 1 | 3 |
| hsa-mir-106a-5p | WEE1 | 1 | 1 | 1 | 3 |
| hsa-mir-106a-5p | NIN | 1 | 1 | 1 | 3 |
| hsa-mir-519d-3p | CEP97 | 1 | 1 | 1 | 3 |
| hsa-mir-106a-5p | ACSL4 | 1 | 1 | 1 | 3 |
| hsa-mir-223-3p | IL6ST | 1 | 1 | 1 | 3 |
| hsa-mir-106a-5p | SOX4 | 1 | 1 | 1 | 3 |
| hsa-mir-106a-5p | ELAVL2 | 1 | 1 | 1 | 3 |
| hsa-mir-223-3p | RHOB | 1 | 1 | 1 | 3 |
| hsa-mir-519d-3p | ZNF280B | 1 | 1 | 1 | 3 |
| hsa-mir-519d-3p | KATNAL1 | 1 | 1 | 1 | 3 |
| hsa-mir-519d-3p | ZBTB18 | 1 | 1 | 1 | 3 |
| hsa-mir-519d-3p | FAM117B | 1 | 1 | 1 | 3 |
| hsa-mir-363-3p | WASL | 1 | 1 | 1 | 3 |
| hsa-mir-206 | UTRN | 1 | 1 | 1 | 3 |
| hsa-mir-519d-3p | MTMR3 | 1 | 1 | 1 | 3 |
| hsa-mir-106a-5p | ANKRD52 | 1 | 1 | 1 | 3 |
| hsa-mir-519d-3p | ZC3H12C | 1 | 1 | 1 | 3 |
| hsa-mir-106a-5p | DCBLD2 | 1 | 1 | 1 | 3 |
| hsa-mir-519d-3p | SMAD5 | 1 | 1 | 1 | 3 |
| hsa-mir-519d-3p | ZBTB7A | 1 | 1 | 1 | 3 |
| hsa-mir-106a-5p | PFKP | 1 | 1 | 1 | 3 |
| hsa-mir-106a-5p | AKAP11 | 1 | 1 | 1 | 3 |
| hsa-mir-106a-5p | SCAMP2 | 1 | 1 | 1 | 3 |
| hsa-mir-106a-5p | SIK1 | 1 | 1 | 1 | 3 |
| hsa-mir-106a-5p | STAT3 | 1 | 1 | 1 | 3 |

**Supplementary Table 6.** lncRNAs combined with miRNAs in ceRNA.

| **lncRNA** | **miRNA** |
| --- | --- |
| IGF2-AS | hsa-mir-503 |
| IGF2-AS | hsa-mir-519d |
| TPTEP1 | hsa-mir-106a |
| TPTEP1 | hsa-mir-137 |
| TPTEP1 | hsa-mir-519d |
| TPTEP1 | hsa-mir-206 |
| TPTEP1 | hsa-mir-223 |
| TPTEP1 | hsa-mir-31 |
| H19 | hsa-mir-519d |
| H19 | hsa-mir-206 |
| C2orf48 | hsa-mir-106a |
| C2orf48 | hsa-mir-519d |
| C2orf48 | hsa-mir-223 |
| MIR31HG | hsa-mir-206 |
| SNHG11 | hsa-mir-106a |
| SNHG11 | hsa-mir-519d |
| SNHG11 | hsa-mir-31 |
| LINC00302 | hsa-mir-31 |
| C20orf197 | hsa-mir-106a |
| C20orf197 | hsa-mir-137 |
| C20orf197 | hsa-mir-519d |
| C20orf197 | hsa-mir-363 |
| KTN1-AS1 | hsa-mir-503 |
| KTN1-AS1 | hsa-mir-106a |
| KTN1-AS1 | hsa-mir-519d |
| LINC00221 | hsa-mir-106a |
| LINC00221 | hsa-mir-519d |
| LINC00221 | hsa-mir-363 |
| LINC00221 | hsa-mir-31 |
| MUC2 | hsa-mir-503 |
| MUC2 | hsa-mir-106a |
| MUC2 | hsa-mir-363 |
| SNHG5 | hsa-mir-363 |
| TDRG1 | hsa-mir-503 |
| TDRG1 | hsa-mir-519d |
| MEG3 | hsa-mir-106a |
| MEG3 | hsa-mir-519d |
| MEG3 | hsa-mir-206 |
| MEG3 | hsa-mir-223 |
| MEG3 | hsa-mir-31 |
| LINC00152 | hsa-mir-503 |
| LINC00152 | hsa-mir-206 |
| LINC00152 | hsa-mir-223 |
| LINC00152 | hsa-mir-31 |
| CLDN10-AS1 | hsa-mir-137 |
| CLDN10-AS1 | hsa-mir-363 |
| MIR181A2HG | hsa-mir-223 |
| EPB41L4A-AS1 | hsa-mir-503 |
| EPB41L4A-AS1 | hsa-mir-106a |
| EPB41L4A-AS1 | hsa-mir-519d |
| EPB41L4A-AS1 | hsa-mir-223 |
| LINC00393 | hsa-mir-106a |
| KCNC4-AS1 | hsa-mir-223 |
| LINC00092 | hsa-mir-206 |
| FGD5-AS1 | hsa-mir-106a |
| FGD5-AS1 | hsa-mir-137 |
| FGD5-AS1 | hsa-mir-519d |
| FGD5-AS1 | hsa-mir-223 |
| FGD5-AS1 | hsa-mir-363 |
| TMEM191A | hsa-mir-503 |
| KIAA0125 | hsa-mir-503 |
| C14orf132 | hsa-mir-503 |
| C14orf132 | hsa-mir-106a |
| C14orf132 | hsa-mir-519d |
| C14orf132 | hsa-mir-206 |
| SOX21-AS1 | hsa-mir-503 |
| LINC00355 | hsa-mir-503 |
| LINC00355 | hsa-mir-223 |
| HCG11 | hsa-mir-519d |
| HCG11 | hsa-mir-31 |
| UBAC2-AS1 | hsa-mir-503 |
| UBAC2-AS1 | hsa-mir-187 |
| PRRT3-AS1 | hsa-mir-363 |
| THAP7-AS1 | hsa-mir-519d |
| THAP7-AS1 | hsa-mir-187 |
| THAP7-AS1 | hsa-mir-223 |
| THAP7-AS1 | hsa-mir-363 |
| THAP7-AS1 | hsa-mir-31 |
| LINC00342 | hsa-mir-31 |
| HOTAIRM1 | hsa-mir-137 |
| HOTAIRM1 | hsa-mir-519d |
| LINC00460 | hsa-mir-503 |
| LINC00460 | hsa-mir-206 |
| GAS5 | hsa-mir-106a |
| GAS5 | hsa-mir-137 |
| GAS5 | hsa-mir-223 |
| GAS5 | hsa-mir-31 |
| LINC00163 | hsa-mir-206 |
| MCF2L-AS1 | hsa-mir-206 |
| TM4SF19-AS1 | hsa-mir-206 |
| BCYRN1 | hsa-mir-503 |
| BCYRN1 | hsa-mir-106a |
| BCYRN1 | hsa-mir-137 |
| BCYRN1 | hsa-mir-519d |
| BCYRN1 | hsa-mir-206 |
| BCYRN1 | hsa-mir-223 |
| BCYRN1 | hsa-mir-363 |
| BCYRN1 | hsa-mir-31 |
| MIR600HG | hsa-mir-137 |
| MIR600HG | hsa-mir-223 |
| MIR600HG | hsa-mir-31 |
| GLIS3-AS1 | hsa-mir-206 |
| DGUOK-AS1 | hsa-mir-223 |
| MLK7-AS1 | hsa-mir-206 |
| SYNPR-AS1 | hsa-mir-363 |
| TRBV11-2 | hsa-mir-363 |
| SNHG3 | hsa-mir-106a |
| SNHG3 | hsa-mir-519d |
| SNHG3 | hsa-mir-206 |
| SNHG3 | hsa-mir-31 |
| SOX2-OT | hsa-mir-206 |
| SOX2-OT | hsa-mir-31 |
| MCCC1-AS1 | hsa-mir-363 |
| ATP1B3-AS1 | hsa-mir-106a |
| LINC00461 | hsa-mir-503 |
| LINC00461 | hsa-mir-106a |
| LINC00461 | hsa-mir-137 |
| LINC00461 | hsa-mir-363 |
| LINC00461 | hsa-mir-31 |
| SNHG6 | hsa-mir-137 |
| SNHG6 | hsa-mir-223 |
| SNHG10 | hsa-mir-106a |
| OIP5-AS1 | hsa-mir-503 |
| OIP5-AS1 | hsa-mir-106a |
| OIP5-AS1 | hsa-mir-137 |
| OIP5-AS1 | hsa-mir-519d |
| OIP5-AS1 | hsa-mir-206 |
| OIP5-AS1 | hsa-mir-223 |
| OIP5-AS1 | hsa-mir-363 |
| PVT1 | hsa-mir-503 |
| PVT1 | hsa-mir-106a |
| PVT1 | hsa-mir-519d |
| PVT1 | hsa-mir-187 |
| PVT1 | hsa-mir-31 |
| LINC00536 | hsa-mir-137 |
| LINC00536 | hsa-mir-519d |
| MALAT1 | hsa-mir-503 |
| MALAT1 | hsa-mir-106a |
| MALAT1 | hsa-mir-519d |
| MALAT1 | hsa-mir-206 |
| MALAT1 | hsa-mir-363 |
| SNHG9 | hsa-mir-31 |
| DIO3OS | hsa-mir-106a |
| DIO3OS | hsa-mir-206 |
| LINC00520 | hsa-mir-503 |
| LINC00520 | hsa-mir-106a |
| LINC00520 | hsa-mir-519d |
| LINC00520 | hsa-mir-223 |
| LINC00520 | hsa-mir-31 |

**Supplementary Table 7. Predictive model construction of clinical features**

| **Race** | **Assignment** |
| --- | --- |
| White | 1 |
| Black | 2 |
| Yellow | 3 |
| Others | 4 |
| **T_Stage** | **Assignment** |
| T1 | 1 |
| T2 | 2 |
| T3 | 3 |
| T4 | 4 |
| **N_Stage** | **Assignment** |
| N0 | 0 |
| N1 | 1 |
| N2 | 2 |
| N3 | 3 |
| **M_Stage** | **Assignment** |
| M0 | 0 |
| M1 | 1 |
| **Stage** | **Assignment** |
| I | 1 |
| II | 2 |
| III | 3 |
| IV | 4 |
